# Effect of the HDAC Inhibitor, Sodium Butyrate, on Neurogenesis in a Rat Model of Neonatal Hypoxia–Ischemia: Potential Mechanism of Action

**DOI:** 10.1007/s12035-019-1518-1

**Published:** 2019-02-14

**Authors:** Joanna Jaworska, Teresa Zalewska, Joanna Sypecka, Malgorzata Ziemka-Nalecz

**Affiliations:** Neurorepair Department, Mossakowski Medical Research Centre, Polish Academy of Sciences, 5 A. Pawinskiego Street, 02-106 Warsaw, Poland

**Keywords:** Neonatal hypoxia–ischemia, Neurogenesis, BDNF–TrkB pathway, CREB, ERK kinase

## Abstract

Neonatal hypoxic–ischemic (HI) brain injury likely represents the major cause of long-term neurodevelopmental disabilities in surviving babies. Despite significant investigations, there is not yet any known reliable treatment to reduce brain damage in suffering infants. Our recent studies in an animal model of HI revealed the therapeutic potential of a histone deacetylase inhibitor (HDACi). The neuroprotective action was connected with the stimulation of neurogenesis in the dentate gyrus subgranular zone. In the current study, we investigated whether HDACi—sodium butyrate (SB)—would also lead to neurogenesis in the subventricular zone (SVZ). By using a neonatal rat model of hypoxia–ischemia, we found that SB treatment stimulated neurogenesis in the damaged ipsilateral side, based on increased DCX labeling, and restored the number of neuronal cells in the SVZ ipsilateral to lesioning. The neurogenic effect was associated with inhibition of inflammation, expressed by a transition of microglia to the anti-inflammatory phenotype (M2). In addition, the administration of SB increased the activation of the TrkB receptor and the phosphorylation of the transcription factor—CREB—in the ipsilateral hemisphere. In contrast, SB administration reduced the level of HI-induced p75^NTR^. Together, these results suggest that BDNF–TrkB signaling plays an important role in SB-induced neurogenesis after HI. These findings provide the basis for clinical approaches targeted at protecting the newborn brain damage, which may prove beneficial for treating neonatal hypoxia–ischemia.

## Introduction

Neonatal hypoxic–ischemia (HI) brain injury is a leading cause of impaired neurodevelopment, resulting in mental retardation, motor dysfunction, and seizures [1–4]. The loss of highly vulnerable axons, oligodendrocyte progenitors, and neurons disrupts maturation of the neural network, with a destructive impact on the structure and connectivity in the brain [5]. Brain damage following hypoxic–ischemic insult is a complex process which develops with a delay during the recovery phase, and by this, it provides an opportunity for therapeutic intervention in the sequence of the intracellular events induced by HI. Unfortunately, there are currently no effective therapies to reduce brain damage and its long-term sequel in infants, despite the progress in knowledge relating the mechanism underlying evolving brain injury. The standard use of therapeutic hypothermia, advised by the International Resuscitation Council as standard of care, reduces the incidence of poor outcome of death or disability only from 66 to 50% [6].

Neurogenesis arising from populations of neural progenitors that persist in neurogenic niches of the subventricular zone (SVZ) and subgranular zone (SGZ) of the dentate gyrus is now an accepted feature of the postnatal and adult mammalian brain [7]. The brain also has been shown to have some capacity for endogenous regeneration after various kinds of insults that lead to the loss of neurons in adult mammalian brains like hypoxia–ischemia, acute seizures, and trauma [8–11]. Studies have also shown that neonatal hypoxic–ischemia induces neural progenitor proliferation followed by the migration of newly generated cells toward the injured brain areas where they acquire the desired phenotype. Furthermore, new cells may integrate into the neuronal network and participate in the recovery from neurological deficits [12, 13].

However, the capacity of endogenous regeneration proved to be rather limited and insufficient for replacing of the lost neurons [13, 14]. Most newly generated neuronal precursors derived from the SVZ neurogenic area do not survive maturation [15]. Therefore, developing strategies aimed at harnessing the neurogenic capacity and repair processes might have significant potential for treating the injured brain. As a result of both in vivo and in vitro studies, it has been established that endogenous neurogenesis can be modulated by various extrinsic factors [16, 17]. One class of drugs with the potential to positively influence post-HI neurogenesis and regeneration is histone deacetylase inhibitors. Histone deacetylase inhibitors (HDACis) are a heterogeneous group of agents that inhibit histone deacetylases (HDACs) and promote posttranslational acetylation of lysine residues within nuclear and cytoplasmic proteins, which may alter their activity and function. The treatment with HDACis results in accumulation of acetylated proteins, which has been shown to either increase gene expression by reducing chromatin compaction or reduce gene activation via increases in repressor transcription [18, 19]. Although acetylation of histones is recognized as a key posttranslational modulation of proteins responsible for the regulation of critical intracellular pathways and are therefore the most intensively investigated substrates, HDACis equally promote the acetylation of several nonhistone proteins, such as transcription factors and signal transduction mediators, determining their interaction, localization, and stability [20].

Some studies have shown HDACis to be neuroprotective and neurogenic in adult ischemia models [18, 21, 22]. However, only a few available reports addressed the effect of HDACis in the hypoxia–ischemia-injured immature brain [23–26]. In our previous work, we found that treatment with sodium butyrate, one of the HDAC inhibitors, administered directly after HI insult, exhibits a neurogenic effect in the SGZ area of the hippocampus. However, despite of this, the endogenous self-repair capacity appeared to be limited, since the more mature neuronal granule cells in DG did not completely recover from experimental HI [26]. In addition, sodium butyrate (SB) treatment did not counteract significantly the functional brain impairments caused by HI [26]. To complete the data related to the effect of HDACi on brain neurogenesis, our present investigation was designed to determine the influence of SB on the stimulation/differentiation of endogenous progenitor cells derived from the neurogenic subventricular zone and thus promotion of neural regeneration in a neonatal rat model of hypoxia–ischemia. It is known that brain injury releases several molecular mediators, many of which encourage neurogenic response.

The increased BDNF level in the damaged hypoxic–ischemic hemisphere, found in our previous study after SB treatment, may imply that trophic support plays a role in the generation of new neural cells. BDNF, as well as NGF, regulates neural cell survival in the developing nervous system. Moreover, they participate in the structural and functional regeneration of nerve tissue after ischemic damage [27–31]. Several findings suggested an important role of BDNF–TrkB receptor signaling in mediating HDACi-induced cell proliferation and differentiation in ischemic adult rodents [32, 33].

BDNF–TrkB signaling activates protein kinases such as PI3K/Akt and ERK/MAPK. Both these pathways converge on transcription factor CREB, of which phosphorylation plays a prominent role in the proliferation, differentiation, and survival of neuronal precursor cells [34, 35]. Furthermore, phosphorylated CREB can enhance the transcription of BDNF and NGF genes and the production of proteins which also stimulate cell survival and differentiation of SVZ progenitor cells and increase the number of newborn cells [36, 37]. Thus, it is tempting to speculate that neurotrophins in conjunction with their receptors might be capable of regulating progenitor proliferation and/or differentiation in the immature brain after neonatal HI.

In an effort to explore the potential underlying mechanism which regulates the neuroprotective/neurogenic response to SB-induced HDAC inhibition, we sought to determine the expression of BDNF and NGF, as well as TrkB receptor and downstream signaling substrates—ERK1/2, Akt, and phosphorylated CREB. In addition, based on the data that HDACi also exerts its effect by promoting acetylation of nonhistone proteins such as those important in microtubule stability, we investigated the effect of HI and SB treatment on the level of alpha-tubulin acetylation. Acetylated alpha-tubulin appears to be important in various cellular processes, including intracellular transport, cell migration, and polarity [38]. Finally, as inflammation is a well-recognized pathogenic factor in perinatal brain injury, we analyzed the microglial cell response to SB treatment 14 days after insult induction, at the time corresponding to the stimulation of neurogenesis after HI.

## Material and Methods

### Experimental Neonatal Hypoxia–Ischemia

All animal experiments were conducted according to regulations following European Union directives. Experimental procedures were approved by the Local Ethics Committee for Animal Experimentation. All efforts were made to minimize the number of animals and their suffering in every step.

Cerebral hypoxia–ischemia was induced in 7-day-old (P7) Wistar rats of either sex by a permanent unilateral common carotid artery ligation, followed by systemic hypoxia [39, 40]. At first, it was determined if there is a sexually dimorphic response to HI and SB treatment. Since no significant differences between male and female rats were noticed, pups of either sex were used in the experiments. As was previously reported, the ligation alone does not decrease cerebral perfusion below critical levels and the addition of hypoxia is required to cause brain infarct [41]. Briefly, pups were anesthetized with isoflurane (4% induction, 2.0% maintenance) carried by O_2_. Once they were fully anesthetized, a midline neck incision was made, and the left common carotid artery was isolated, double ligated with surgical silk, and cut between two ligatures. The incision was then sutured with monofilament nylon. Sham-operated animals underwent the same surgical procedure without the ligation of the carotid artery. The time length of anesthesia lasted on average 5 min. After surgery, the rat pups were returned to their home cage for 1 h recovery. Hypoxia was induced by placing the animals in a chamber (35 °C) and subjecting them to a mixture of 7.6% oxygen in nitrogen for 1 h. After the hypoxic exposure, the pups were returned to their dams. They were reared at 20 °C environmental temperature with a light:dark cycle of 12:12 h and food and water were made available ad libitum*.*

Age-matched sham-operated animals served as controls. Pups from each litter were randomly assigned to four experimental groups (five rats per group and time point): (1) control group (vehicle treatment), (2) control animals (SB treatment), (3) animals which underwent HI (vehicle treatment), and (4) animals which underwent HI (SB treatment). Animals were sacrificed at specific time points—1, 3, 6, 7, 9, 11, 14, or 28 days—after the injury.

### Drug Administration and Bromodeoxyuridine Labeling

#### Drug Treatment

Rats subjected to HI or sham-operated were treated with subcutaneous injections of SB (Sigma-Aldrich; cat. no. B5887, in a dose 300 mg/kg body weight) or vehicle (saline) starting immediately after hypoxic exposure and lasting five consecutive days.

#### Bromodeoxyuridine Labeling

**Fig. 1:**
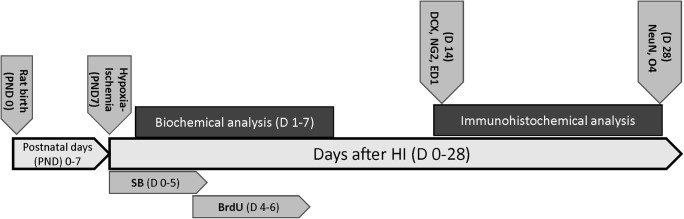
Diagram of experiments design in the present study. Cerebral hypoxia-ischemia was induced in 7-day-old (PND 7) Wistar rats. Rats subjected to HI or sham-operated were treated with subcutaneous injections of sodium butyrate (300 mg/kg body weight) or vehicle (saline) starting immediately after hypoxic exposure and lasting 5 consecutive days. All animals dedicated to immunohistochemical studies received BrdU injections twice daily for 3 consecutive days starting at 4 days after HI. Animals in this group were perfused transcardially with 4% PFA 14 and 28 days after the insult. Biochemical analysis was performed on non-perfused brains (1, 3, and 7  days after HI)

Endogenous cell proliferation was determined by 5-bromo-2-deoxyuridine (BrdU) cell incorporation. BrdU (Sigma-Aldrich, cat. no. B9285) was administered intraperitoneally (50 mg/kg per injection, in sterile 0.9% NaCl plus 0.007 N NaOH). Two-injection paradigms were used. In the first paradigm, animals received a single dose of BrdU and were sacrificed 24 h after the injection. This procedure was used to determine the number of cells that incorporated BrdU during a 24-h period at a specific time point after HI (3, 6, 9, 11, 14 days). This allowed to find the time of the most intensive cell proliferation after HI and to choose the best window of BrdU administration in the next step of the study. To determine the phenotype of newborn cells, animals received BrdU injections twice daily (12 h apart) for three consecutive days starting 4 days after the onset of hypoxia–ischemia. Animals in this group were sacrificed 14 and 28 days after the insult. Animal treatment and tissue collection are summarized in Fig. 1.

### Tissue Preparation

For immunohistochemical studies, as well as for histological staining, animals (14 and 28 days after the insult) were deeply anesthetized with 100 mg/kg body weight ketamine combined with 10 mg/kg body weight xylazine, perfused transcardially first with phosphate buffered saline (PBS) followed by a fixative solution (4% paraformaldehyde, PFA, in 0.1 M phosphate buffer, pH 7.4). The brains were removed and submerged in the same fixative solution for 3 h at 4 °C. Following postfixation, the brains were cryoprotected overnight in 30% sucrose solution (in 0.1 M PBS), frozen rapidly on dry ice, and placed in − 80 °C storage. Coronal cryostat sections of the brain (30 μm thick) were cut on the cryostat at the level of the lateral ventricles (in serial order to create 10 series sections) and used for double immunofluorescence or histological staining.

Biochemical analysis was performed on nonperfused brains. Deeply anesthetized animals were sacrificed through decapitation (1, 3, and 7 days after HI). Isolated brains were dissected on two hemispheres—ipsilateral/ischemic and contralateral/control—immediately frozen on dry ice, and stored at − 80 °C until use.

### Brain Injury Evaluation

Brain damage was evaluated neuropathologically by hematoxylin–eosin (HE) staining 14 days after the insult (at postnatal day 21). Brain sections were mounted on slides coated with 2% silane solution (Sigma-Aldrich, cat. no. A3648) in acetone. Then the sections were stained in hematoxylin (Sigma-Aldrich, cat. no. H3136) for 6 min and then washed in running tap water for 30 min and stained in 1% Eosin Y (Sigma-Aldrich, cat. no. E4009) for 2 min. After this step, the slides were dehydrated in increasing concentration of ethanol alcohols (70, 96, and 100% each 3 min) and cleared in xylene (2 × 3 min). Finally, they were mounted in DPX mounting medium (Sigma-Aldrich, cat. no. 06522), coverslipped, and examined using light microscopy.

### Immunohistochemical Staining

Immunohistochemistry was performed on 30 μm free-floating cryostat sections of the lateral ventricle formation. The following antibodies were used (source, catalog number, and final dilution): sheep polyclonal anti-BrdU (Abcam, cat. no. ab1893, 1:500), mouse monoclonal anti-BrdU (Santa Cruz Biotechnology, cat. no. sc-32323, 1:100), rabbit polyclonal anti-doublecortin (DCX; Cell Signaling, cat. no. 4604, 1:200), mouse monoclonal anti-NeuN (Millipore, cat. no. MAB377, 1:200), rabbit polyclonal anti-NG2 chondroitin sulfate proteoglycan (Millipore, cat. no. AB5320, 1:200), mouse monoclonal anti-oligodendrocyte marker (O4; Millipore, cat. no. MAB345, 1:200), mouse monoclonal anti-ED1 (CD68) (AbD Serotec, cat. no. MCA341R, 1:100), goat polyclonal anti-Arg-1 (arginase-1) (Santa Cruz, cat. no. sc-18354, 1:250), and rabbit polyclonal anti-IL-1β (Santa Cruz, sc-7884, 1:250).

For detection of BrdU incorporation, DNA was first denaturated by incubation of sections with 2 N HCl at 37 °C for 1 h and rinsed for 15 min in 0.1 M sodium tetraborate (0.2 M H_3_BO_3_, 0.05 M Na_2_B_4_O_7_∙10H_2_O, pH 8.4) at room temperature (RT). After blocking with 10% normal goat/donkey serum (Sigma-Aldrich, S26-M/D9663) in PBS containing 0.25% Triton X-100 (Sigma-Aldrich, T8787) for 60 min and washing with PBS (3 × 5 min) at RT, sections were incubated with anti-BrdU overnight at 4 °C. Following the washing procedure (3 × 5 min PBS, RT), the primary antibodies were revealed by appropriate secondary FITC-conjugated antibodies 60 min at RT and in the dark.

Differentiation of BrdU-positive cells was monitored with markers labeling neurons and oligodendrocytes at various stages of maturation (DCX, NeuN, NG2, O4), as well as activated microglia (ED1, Arg-1, IL-1β). After BrdU staining, the brain tissue sections were rinsed in PBS (3 × 5 min, RT) and incubated with primary antibodies overnight at 4 °C. The following day, the sections were washed in PBS (3 × 5 min, RT) and exposed to secondary appropriate Cy3-conjugated secondary antibodies for 1 h at room temperature. After final washing in PBS (3 × 5 min, RT), sections were mounted on slides and coverslipped using DAKO fluorescent mounting medium (Dako, S3023). In all cases, negative controls were processed in the same manner on adjacent sections but with the primary antibodies omitted.

Double labeling was verified using a confocal laser scanning microscope (LSM 780, Carl Zeiss, Germany) applying a ×10 or ×20 objective and crop function with ZEN software. A helium–neon laser (543 nm) was utilized in the excitation of Alexa Fluor 546 (Cy3), and an argon laser (488 nm) was applied in the excitation of FITC. Every section was evaluated using a computerized system, and the positive cells were displayed on a computer screen. All counting was performed using ImageJ 1.46 software. The number of positive labeled cells as well as fluorescence intensity was assessed in an average of five brain sections per animal in an area of 1.44 mm^2^.

### Quantitative Measurement of NGF Protein Concentration

To estimate the amount of NGF in lysates obtained from both brain hemispheres, the ChemiKine Nerve Growth Factor Sandwich ELISA (Millipore, cat. no. CYT304) test was applied according to the supplier’s instructions. After performing the Sandwich ELISA assays, the plates were read at 450 nm using a spectrophotometric plate reader Fluorostar Omega (BMG LabTech).

### Western Blot Analysis

The following antibodies (source, catalog number, and final dilution) were used for analysis: rabbit monoclonal anti acetyl-alpha-tubulin (Lys40) (Cell Signaling, cat. no. 5335, 1:1000), mouse monoclonal anti-pro-BDNF (Santa Cruz, cat. no. sc-65514, 1:1000), rabbit polyclonal anti-TrkB (Santa Cruz, cat. no. sc-12, 1:200), rabbit polyclonal anti-phospho-TrkB (pTyr515) (Thermo Fisher, cat. no. PA5-36695, 1:200), rabbit monoclonal anti-p75 (Cell Signaling, cat. no. 8238, 1:500), mouse monoclonal anti-phospho-CREB (Ser133) (Millipore, cat. no. 05-807, 1:500), rabbit polyclonal anti-ERK1/2 (Cell Signaling, cat. no. 9102, 1:1000), mouse monoclonal anti-phospho-ERK1/2 (Thr202/Tyr204) (Cell Signaling, cat. no. 9106, 1:1000), rabbit polyclonal anti-Akt (Cell Signaling, cat. no. 9272, 1:1000), rabbit polyclonal anti-phospho-Akt (Ser473) (Cell Signaling, cat. no. 9271, 1:1000), and mouse monoclonal anti-actin (MP Biomedicals, cat. no. 0869100 MP, 1:500).

Brain tissues were homogenized in RIPA lysis buffer (10 mM Tris–HCl, pH 7.5 containing 150 mM NaCl, 1% Nonidet P40, 0.1% SDS, 1% Triton X-100, PMSF 0.1 mg/ml) and a proteinase and phosphatase inhibitor cocktail (Life Technologies, 1:100). Lysates were clarified by centrifugation at 13,000*g* for 10 min at 4 °C. The supernatant was collected and used for analysis of acetyl-alpha-tubulin, pro-BDNF, TrkB, phospho-TrkB, p75^NTR^, ERK, phospho-ERK, Akt, phospho-Akt, and phosho-CREB. Protein concentrations in the supernatants were determined using a Bio-Rad DCTM protein assay kit (Bio-Rad, cat. no. 5000112). Samples (50 μg protein) were ran on 10–15% SDS-PAGE gels and transferred onto nitrocellulose membranes (GE Healthcare Life Sciences, Amersham™ Protran™ supported 0.45 μm NC, cat. no. 10600018). After blocking, membranes were probed with specific primary antibodies and then incubated with appropriable horseradish peroxidase-conjugated secondary IgG antibodies (Sigma-Aldrich). Immunoblot signals were visualized using ECL chemiluminescence kit (GE Healthcare Life Sciences, cat. no. RPN2106). The chemiluminescent reaction was detected by membrane exposition to an X-ray Hyperfilm™ ECL film (GE Healthcare Life Sciences, cat. no. 70487). To verify specific reactions, the same procedure was used with the omission of the primary antibody, and to verify an equal loading of protein per line, the beta-actin antibody was used as an internal control for each immunoblotting. Semiquantitative evaluation of protein levels detected by immunoblotting was performed by computer-assisted densitometric scanning (LKB Utrascan XL, Program GelScan). The level of protein immunoreactivity was determined by frequent analysis of multiple immunoblots.

### Statistical Analysis

The GraphPad PRISM 5.0 software was used for the statistical analysis of the received data. One-way analysis of variance (ANOVA) followed by Bonferroni’s multiple comparison test was done for comparison among all experimental groups. All values were expressed as mean ± SD. The data were considered significant at *p* value < 0.05.

## Results

### Brain Damage After HI: Effect of SB

**Fig. 2:**
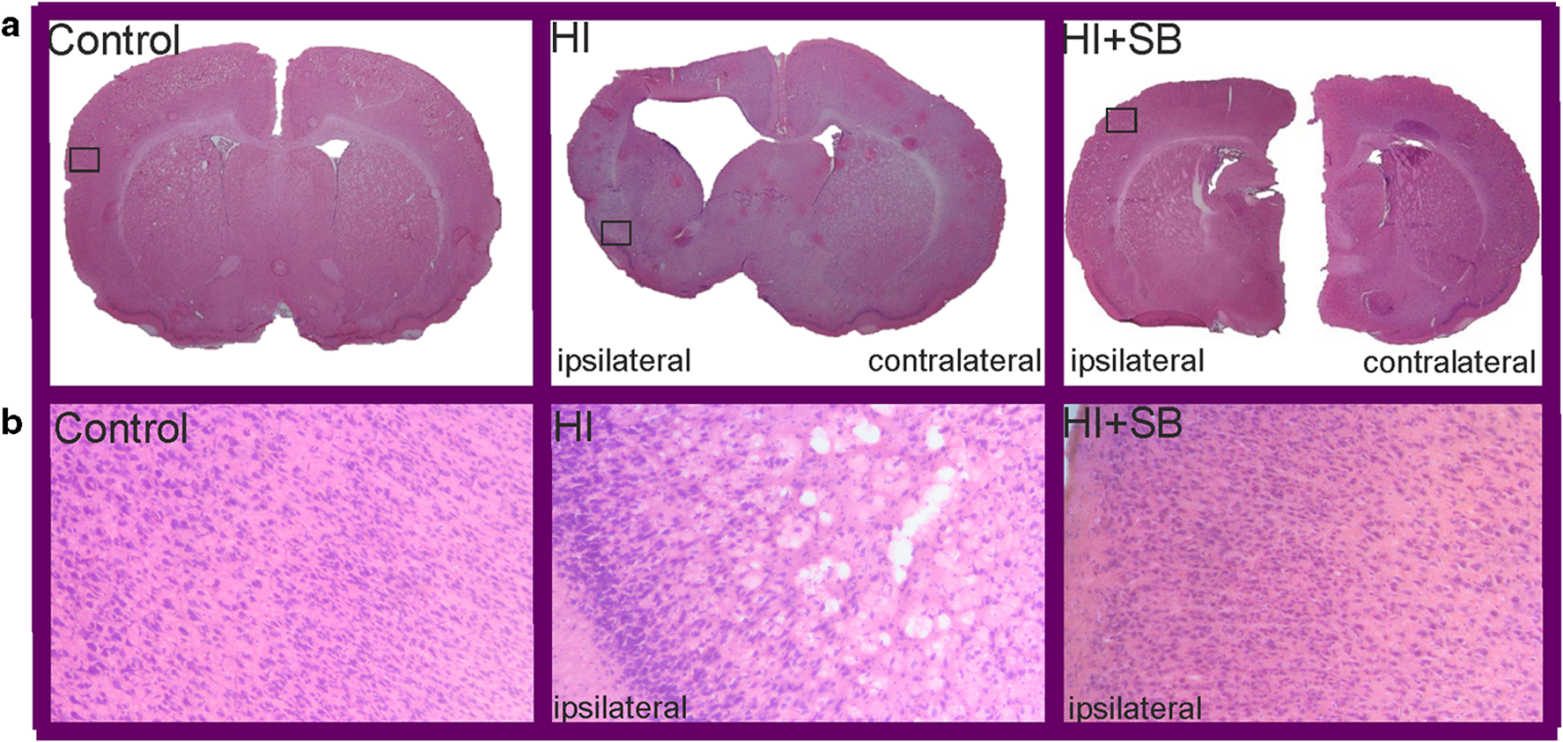
Sodium butyrate treatment reduces hypoxia–ischemia-induced brain damage in neonates. Seven-day-old rats were subjected to hypoxia–ischemia followed by 14 days of recovery. SB or vehicle was administered directly after the onset of HI and for five consecutive days. **a** Brain coronal sections from sham control animals and from animals 14 days after HI (with or without SB treatment) stained with hematoxylin–eosin (HE). **b** Lower panel represents magnification (×100) of the ipsilateral hemisphere area (marked with rectangles in the upper panel). Note the loss of neurons and signs of cerebral edema in the cortex of the ipsilateral hemisphere. Sodium butyrate administration provided neuroprotection in comparison with nontreated animals. Photomicrographs are representative of observations made from five animals per group

Both the left and right hemispheres of all rats (sham control, HI with or without SB treatment) were subjected to histological evaluation at 14 days after the insult (P21). Coronal sections (cut at the level of the lateral ventricles) stained with HE present loss of neurons and signs of cerebral edema with swollen cells throughout the ipsilateral cortex exclusively, in agreement with our previous report [26]. In most animals (80%), the SVZ was spared histologically. However, in 20% of cases, we observed ventricular enlargement (Fig. 2). In consequence, the SVZ adjacent to the ventricular margin became narrower in comparison with controls. Moreover, in these animals, the subventricular zone did not exhibit a clear dorsolateral tail beneath the corpus callosum, which is typically seen in intact brains. No ventriculomegaly was noted in the contralateral hemisphere as well as in the brains after SB treatment.

### Time Course of Cell Proliferation in the SVZ

**Fig. 3:**
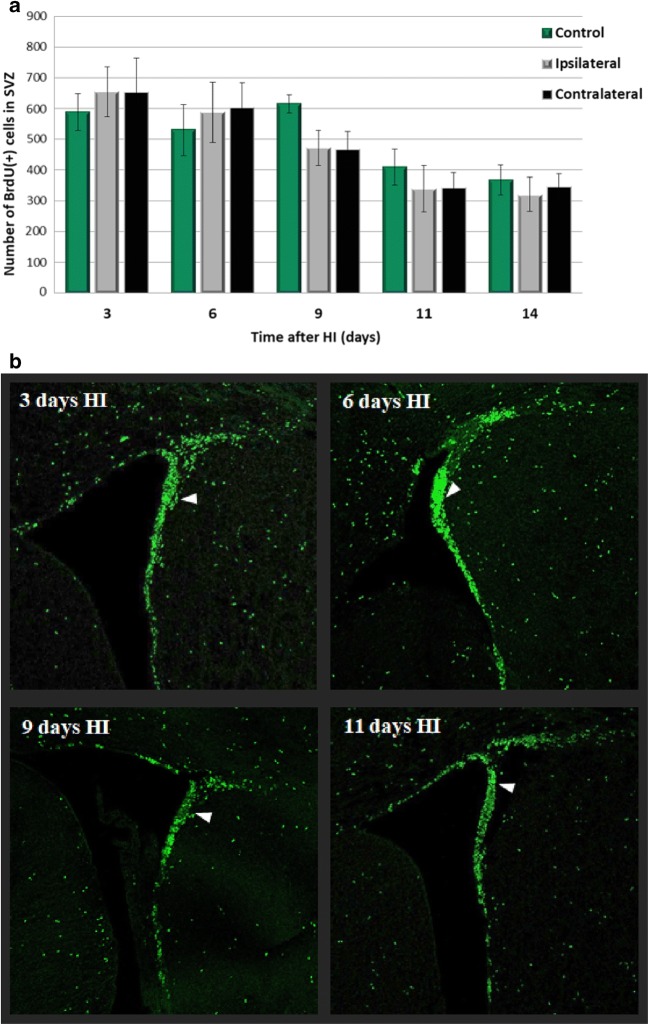
Time course of cell proliferation in the SVZ. Animals received a single dose of BrdU (50 mg/kg) 24 h prior to sacrifice, at 3, 6, 9, 11, and 14 days after HI, and the brains were processed for BrdU immunohistochemistry. **a** The number of BrdU-labeled nuclei within the SVZ area in sham-operated control animals and at different times after ischemia in both sides: injured ipsilateral and noninjured contralateral. The values are mean ± SD of five animals per group and time point. One-way ANOVA and Bonferroni test did not indicate significant differences in BrdU labeling between control (*green bars*), ipsilateral (*gray bars*), and contralateral SVZ (*black bars*) in investigated time points. **b** Confocal photomicrographs show newly divided cells in the control group and after HI (*arrowheads*). While the greatest number of BrdU-positive cells is seen in SVZ, their number is also pronounced in the striatum

The time course of cell proliferation in the neurogenic SVZ was studied at specific time points after hypoxic–ischemic injury (3, 6, 9, 11, and 14 days). For this purpose, animals received a single dose of BrdU 24 h prior to sacrifice. The number of newly generated cells was determined in the entire SVZ region by monitoring the incorporation and subsequent immunohistochemical detection of BrdU. Confocal analysis of brain sections revealed immunoreactive cells concentrated in SVZ; however, some stained cells were also distributed throughout the adjacent corpus callosum and striatum. The highest density of BrdU-positive cells within the SVZ area was detected between 3 and 6 days of recovery, similar to our previous studies performed in the SGZ region [26]. Thereafter, cell proliferation tended to decrease in all experimental groups by about 40% in average as compared with the primary time point, indicating lowering of the dynamic of stem/progenitor cell proliferation (Fig. 3). As evidenced by the analysis of brain sections, there was no difference in the number of proliferating cells (BrdU+) between injured and control, sham-operated animals. Moreover, the exposure to SB did not affect the number of BrdU-positive cells in the SVZ—neither in the ipsi- nor in the contralateral side (not shown).

### Phenotypic Characterization of Proliferating Cells in the Subventricular Zone After Neonatal Hypoxia–Ischemia

**Fig. 4:**
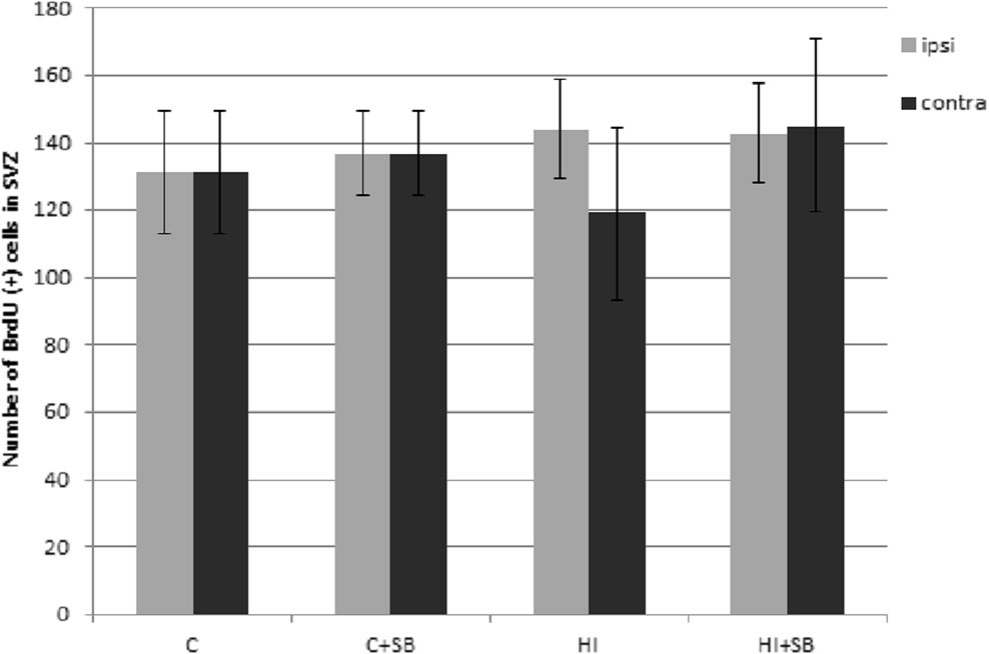
Sodium butyrate has no effect on cell proliferation in SVZ after HI. Neonatal HI was induced at postnatal day 7. SB was administered directly after the onset of HI and for five consecutive days. BrdU was administered daily for three consecutive days starting 4 days after the onset of HI. Animals were sacrificed 14 days after the insult. Graph shows the number of BrdU-labeled nuclei within the SVZ of control and HI animals with or without SB treatment. The values are mean ± SD of five animals per experimental group. One-way ANOVA and Bonferroni test did not indicate significant differences in the number of BrdU-labeled nuclei within the SVZ area between control and HI animals, independent of SB treatment

For the purpose of these studies, animals received multiple BrdU injections on days 4–6 after hypoxia–ischemia and were sacrificed at 14 or 28 days of recovery. At first, we investigated whether in this experimental paradigm SB will influence cell proliferation in the SVZ. Quantitative analysis of BrdU labeling in tissue samples obtained 2 weeks after injury indicated that the number of cells incorporating BrdU in the hypoxic–ischemic SVZ (ipsilateral) was comparable with the hypoxic only (contralateral side) as well as with the age-matched sham-operated control (Fig. 4). Also, treatment with SB exerted no effect on the number of dividing cells (BrdU-positive), compared with the sham group.

To further characterize the fate of newly arisen cells, brain tissue sections from sham and HI rats with or without SB treatment were double-stained for BrdU and different neural antigens—DCX (for neuroblasts), NeuN (for mature neurons), NG2 (detected in oligodendrocyte progenitor cells), O4 (for immature, nonmyelinating oligodendrocytes), and ED1 (for microglia).

**Fig. 5:**
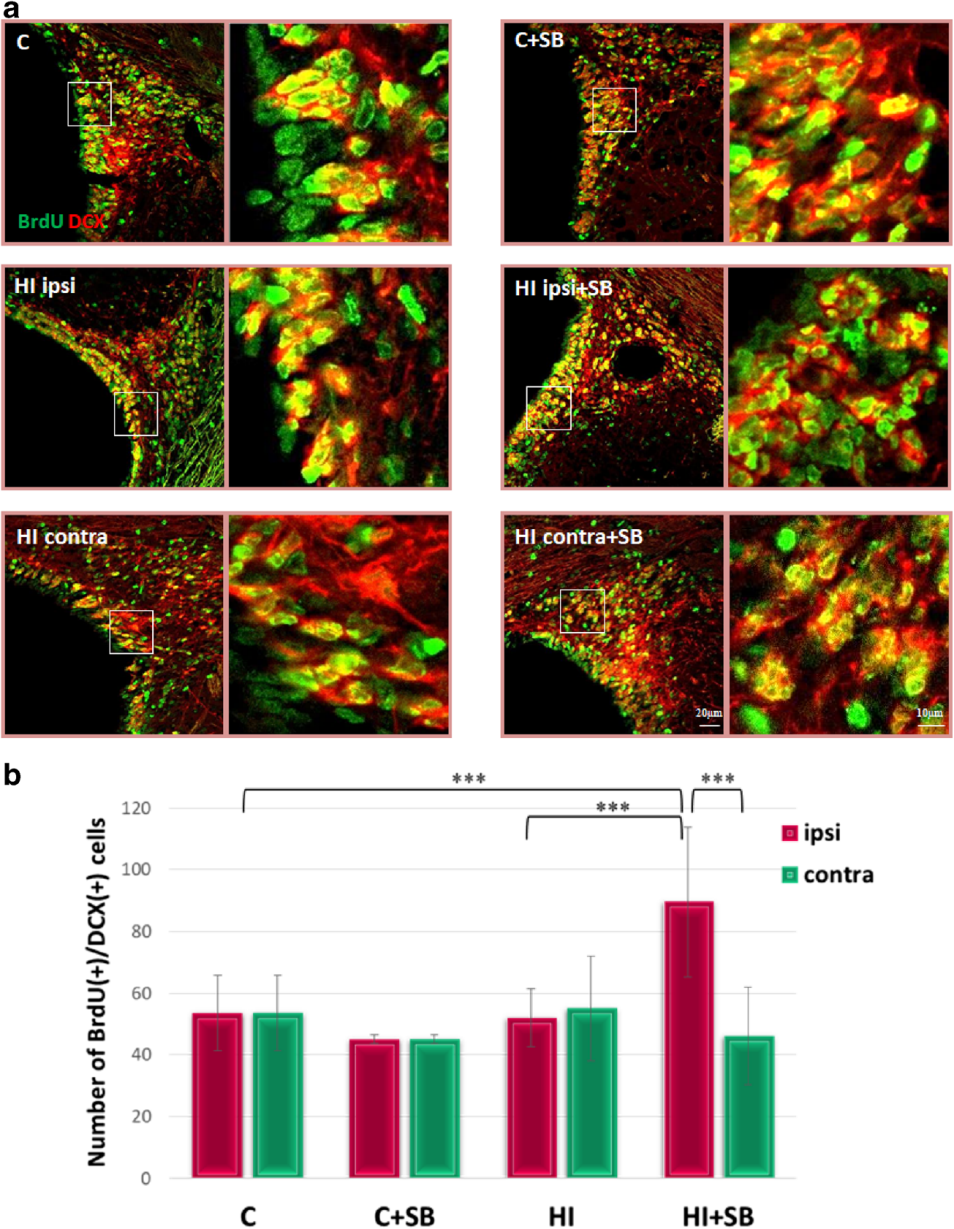
Sodium butyrate promotes generation of new neuroblasts in the SVZ after HI. Neonatal HI was induced at postnatal day 7. SB was administered directly after the onset of HI and for five consecutive days. BrdU was administered daily for three consecutive days starting 4 days after the onset of HI. Brain sections from control animals and from animals 14 days after HI were double-labeled with anti-BrdU (*green*) and anti- DCX (*red*) antibodies. **a** Confocal photomicrographs show double-labeled cells in SVZ of control and HI animals 14 days after the insult with or without SB treatment. Enlargements present areas marked in rectangles. **b** Number of BrdU/DCX-positive cells quantified in the SVZ area (0.36 mm^2^). The values are mean ± SD from five animals per experimental group. One-way ANOVA and Bonferroni test indicate significant differences between investigated groups, ***p < 0.001. Abbreviations: *C*—control, *ipsi*—ipsilateral, *contra*—contralateral

To evaluate if proliferating cells progressed to form neural progenitors, BrdU-labeled cells were stained with DCX, a marker specific for neuroblasts (immature neurons). Double fluorescent studies revealed numerous BrdU-positive nuclei in the neurogenic SVZ closely associated with neuronally committed precursors and/or immature neurons expressing microtubule-associated protein—DCX. Representative photomicrographs of the immunohistochemistry and respective quantitative data are shown in Fig. 5. Surprisingly, and conversely to the previously reported response to HI in the subgranular zone [26], the same experimental conditions did not alter the quantity of BrdU/DCX-labeled cells distributed extensively within the SVZ area at 14 days of recovery. The only notable change was a marked increase in the number of neuroblasts in the ipsilateral hypoxic–ischemic hemisphere after SB administration compared either to HI nontreated with HDACi or to control (*p* < 0.001).

**Fig. 6:**
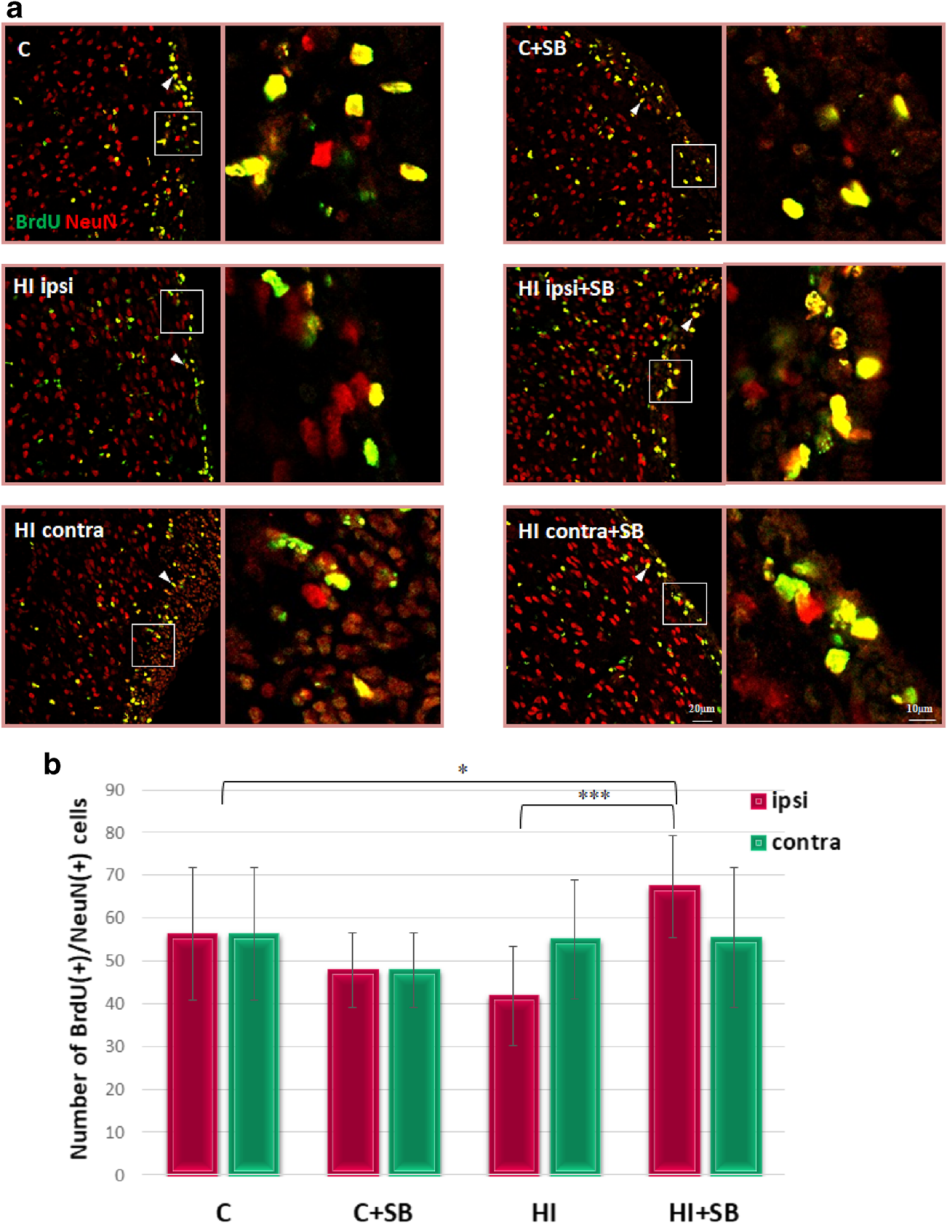
Sodium butyrate increases the number of newly generated neurons in the SVZ after HI. Neonatal HI was induced at postnatal day 7. SB was administered directly after the onset of HI and for five consecutive days. BrdU was administered daily for three consecutive days starting 4 days after the onset of HI. Brain sections from control animals and from animals 28 days after HI were double-labeled with anti-BrdU antibody (*green*) and anti-NeuN, neuronal cell marker (*red*). **a** Confocal photomicrographs show double-labeled cells in the SVZ of control and HI animals 28 days after the insult with or without SB treatment (*arrowheads*)*.* Enlargements present areas marked in rectangles. **b** Number of BrdU/NeuN-positive cells quantified in the SVZ area (0.36 mm^2^). The values are mean ± SD from five animals per experimental group. One-way ANOVA and Bonferroni test indicate significant differences between investigated groups, *p < 0.05; ***p < 0.001. Abbreviations: *C*—control, *ipsi*—ipsilateral, *contra*—contralateral

To examine whether neuroblasts localized in the SVZ differentiate to mature neurons, we performed double labeling assay using BrdU with NeuN, a marker of mature neuronal cells. Quantified results are shown in Fig. 6. It clearly appears that 28 days after hypoxic–ischemic injury, the number of double-stained BrdU/NeuN cells in the SVZ area (ipsi- and contralateral) remained close to the control. However, a detailed analysis of the several stained slices indicated a tendency of the new cells (BrdU/NeuN-positive) to decrease in the ipsilateral side. Exposure to SB resulted in a significant increase in the number of newly formed neurons in the SVZ of injured hemisphere as compared to the untreated animals (*p* < 0.001) or age-matched control (*p* < 0.05). This finding provided evidence that SB treatment increased the generation of new neurons in the SVZ of the HI hemisphere.

**Fig. 7:**
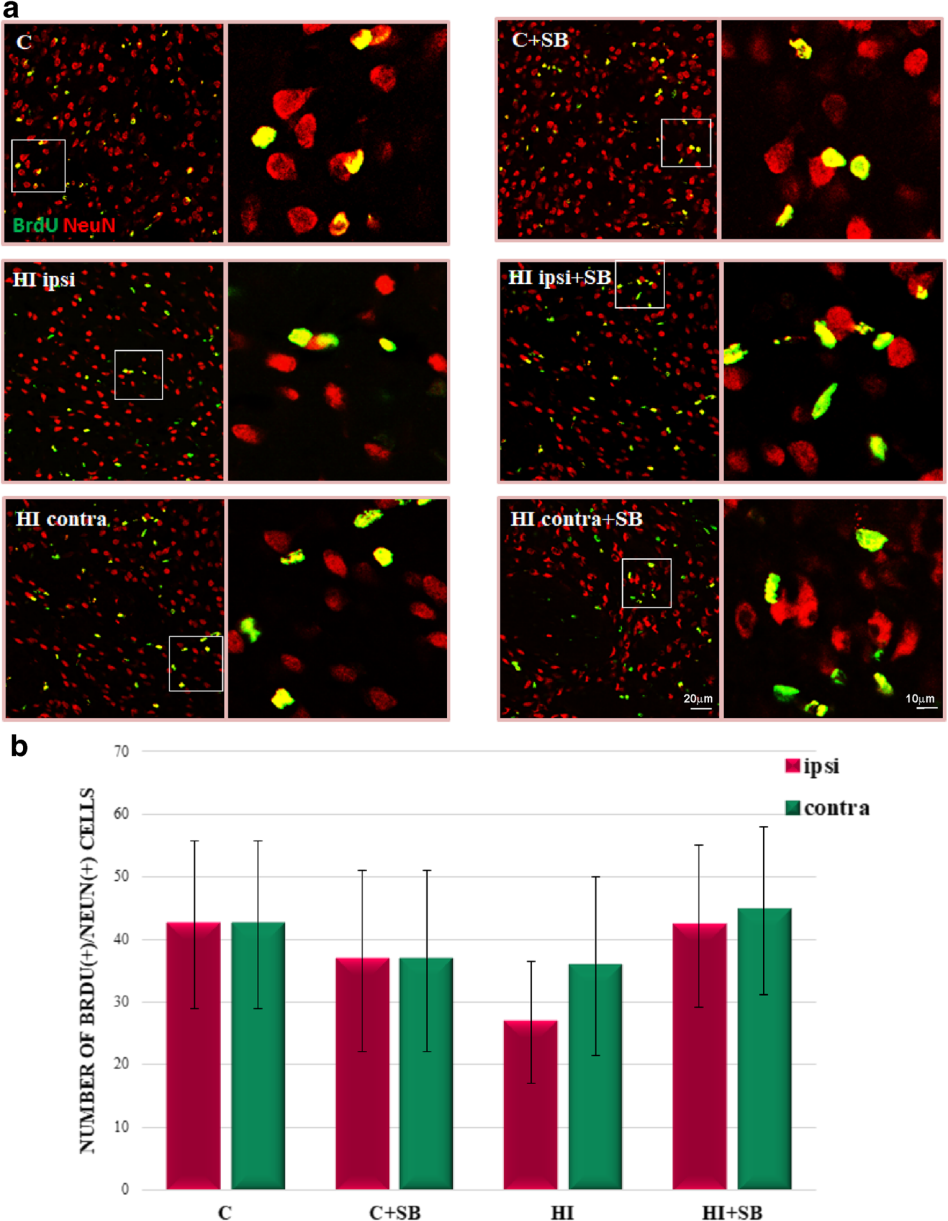
Sodium butyrate does not influence the number of newly generated neurons in the striatum after HI. Neonatal HI was induced at postnatal day 7. SB was administered directly after the onset of HI and for five consecutive days. BrdU was administered daily for three consecutive days starting 4 days after the onset of HI. Brain sections from control animals and from animals 28 days after HI with or without SB treatment were double-labeled with anti-BrdU antibody (*green*) and anti-NeuN, neuronal cell marker (*red*). **a** Confocal photomicrographs show double-labeled cells in the striatum of control and HI animals 28 days after the insult with or without SB treatment (*yellow*)*.* Enlargements present areas marked in rectangles. **b** Number of BrdU/NeuN-positive cells quantified in the striatum area (0.36 mm^2^). The values are mean ± SD from five animals per group. One-way ANOVA and Bonferroni test did not indicate significant differences between investigated groups. Abbreviations: *C*—control, *ipsi*—ipsilateral, *contra*—contralateral

In addition, it is worth pointing out that immunofluorescent microscopy analysis elicited a consistently low level of DCX-positive cells in the striatum with only single cells presenting co-staining with BrdU, mostly placed in the area adjacent to the SVZ (not shown). Furthermore, while double-stained newly generated neurons (BrdU/NeuN) were generally found in the SVZ area, a subset of these cells was also dispersed in the whole striatum; however, their number estimated in all experimental groups was maintained on a similar level in both investigated hemispheres (Fig. 7).

**Fig. 8:**
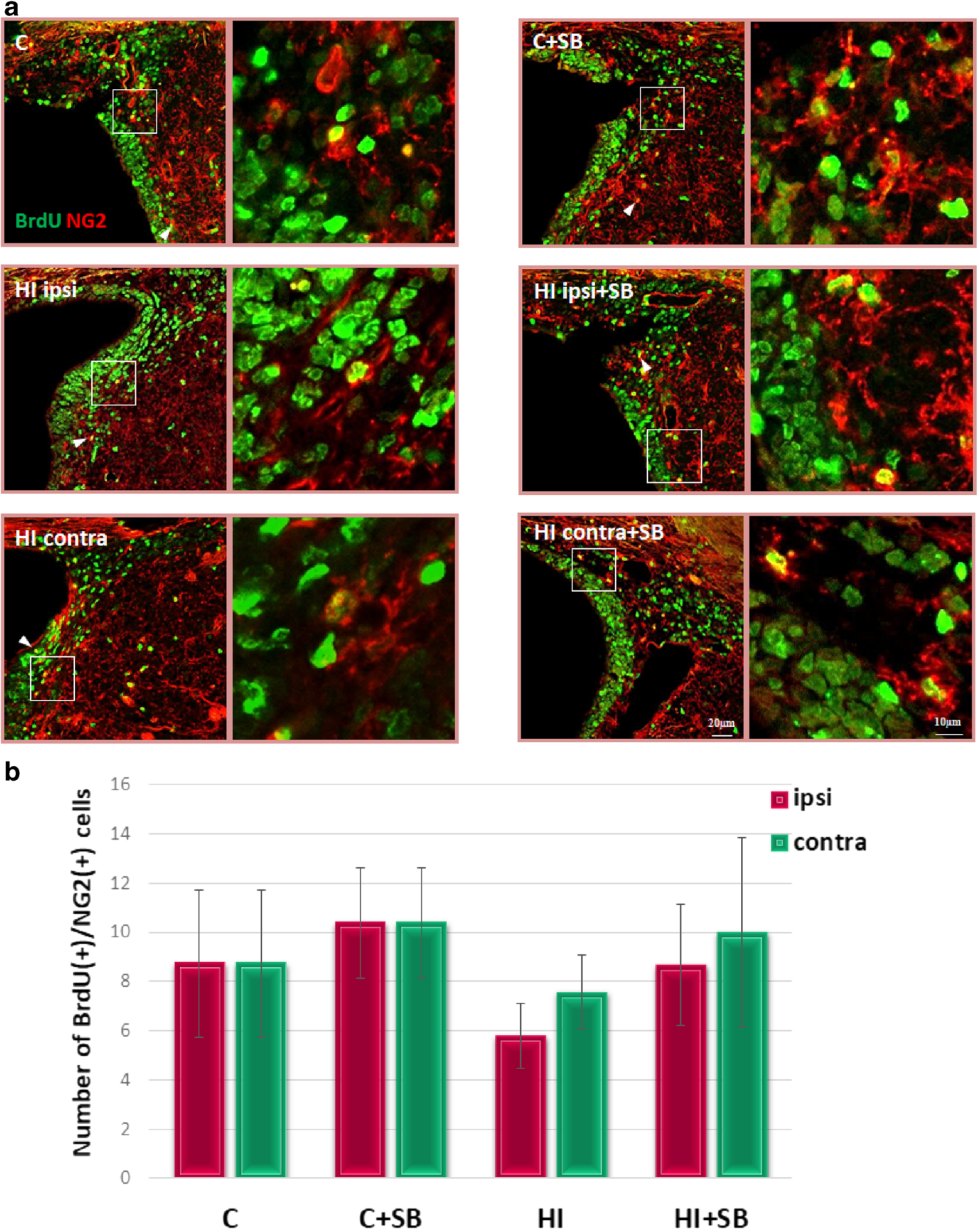
Sodium butyrate does not increase the number of oligodendrocyte progenitors in the SVZ after HI. Neonatal HI was induced at postnatal day 7. SB was administered directly after the onset of HI and for five consecutive days. BrdU was administered daily for three consecutive days starting 4 days after the onset of HI. Brain sections from control animals and from animals 14 days after HI were double-labeled with anti-BrdU antibody (*green*) and the oligodendrocyte precursor cell marker—NG2 (*red*). **a** Confocal photomicrographs show double-labeled cells in the SVZ of control and HI animals 14 days after the insult with or without SB treatment (*arrowheads*). Enlargements present areas marked in rectangles. **b** Number of BrdU/NG2-positive cells quantified in the SVZ area (0.36 mm^2^). The values are mean ± SD from five animals per group. One-way ANOVA and Bonferroni test did not indicate significant differences between experimental groups. Abbreviations: *C*—control, *ipsi*—ipsilateral, *contra*—contralateral

**Fig. 9:**
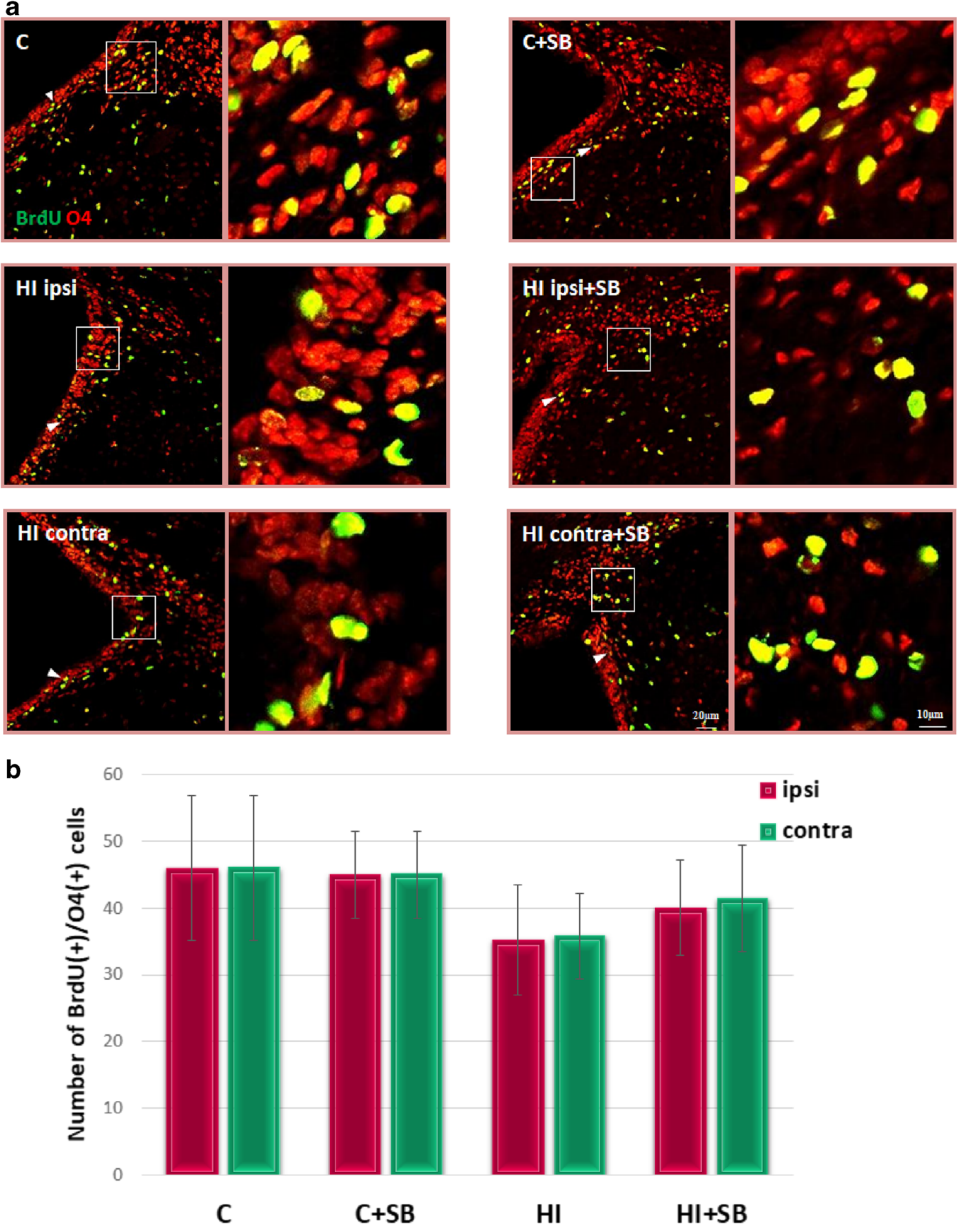
Sodium butyrate does not influence the number of nonmyelinating oligodendrocytes expressing O4 in the SVZ after HI. Neonatal HI was induced at postnatal day 7. SB was administered directly after the onset of HI and for five consecutive days. BrdU was administered daily for three consecutive days starting 4 days after the onset of HI. Brain sections from control animals and from animals 28 days after HI were double-labeled with anti-BrdU antibody (*green*) and the nonmyelinating oligodendrocyte marker—O4 (*red*). **a** Confocal photomicrographs show double-labeled cells in the SVZ of control and HI animals 28 days after the insult with or without SB treatment (*arrowheads*)*.* Enlargements present areas marked in rectangles. **b** Number of BrdU/O4-positive cells quantified in the SVZ area (0.36 mm^2^). The values are mean ± SD from five animals per group. One-way ANOVA and Bonferroni test did not indicate significant differences in the number of double-labeled cells between the investigated groups. Abbreviations: *C*—control, *ipsi*—ipsilateral, *contra*—contralateral

**Fig. 10:**
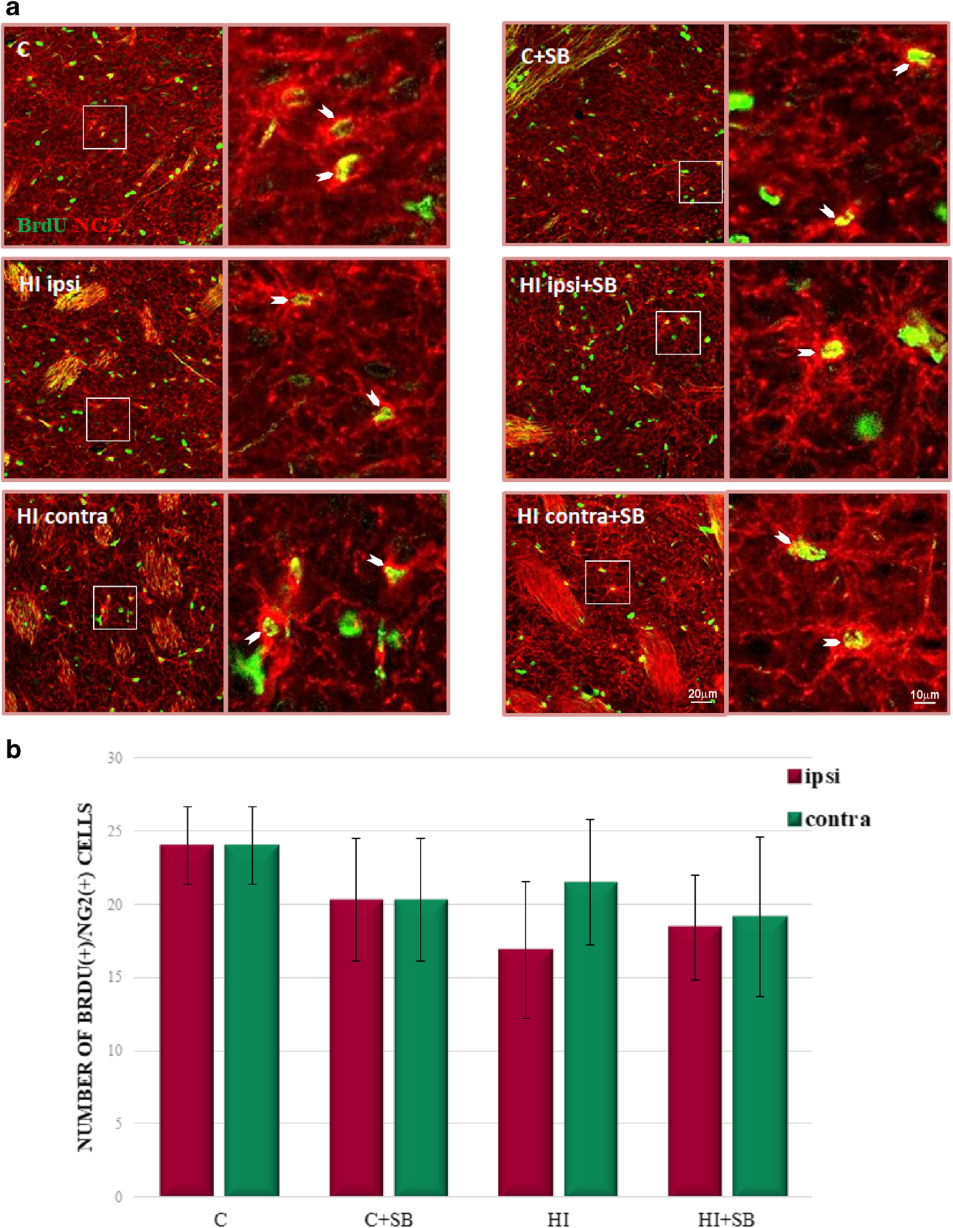
Sodium butyrate does not influence the number of oligodendrocyte progenitors in the striatum. Neonatal HI was induced at postnatal day 7. SB was administered directly after the onset of HI and for five consecutive days. BrdU was administered daily for three consecutive days starting 4 days after the onset of HI. Brain sections from control animals and from animals 14 days after HI were double-labeled with anti-BrdU antibody (*green*) and the oligodendrocyte precursor cell marker—NG2 (*red*). **a** Confocal photomicrographs show double-labeled cells in the striatum (*arrowheads*)*.* Enlargements present areas marked in rectangles. **b** Number of BrdU/NG2-positive cells quantified in the SVZ area (0.36 mm^2^). The values are mean ± SD from five animals per group. One-way ANOVA and Bonferroni test did not indicate significant differences between experimental groups. Abbreviations: *C*—control, *ipsi*—ipsilateral, *contra*—contralateral

**Fig. 11:**
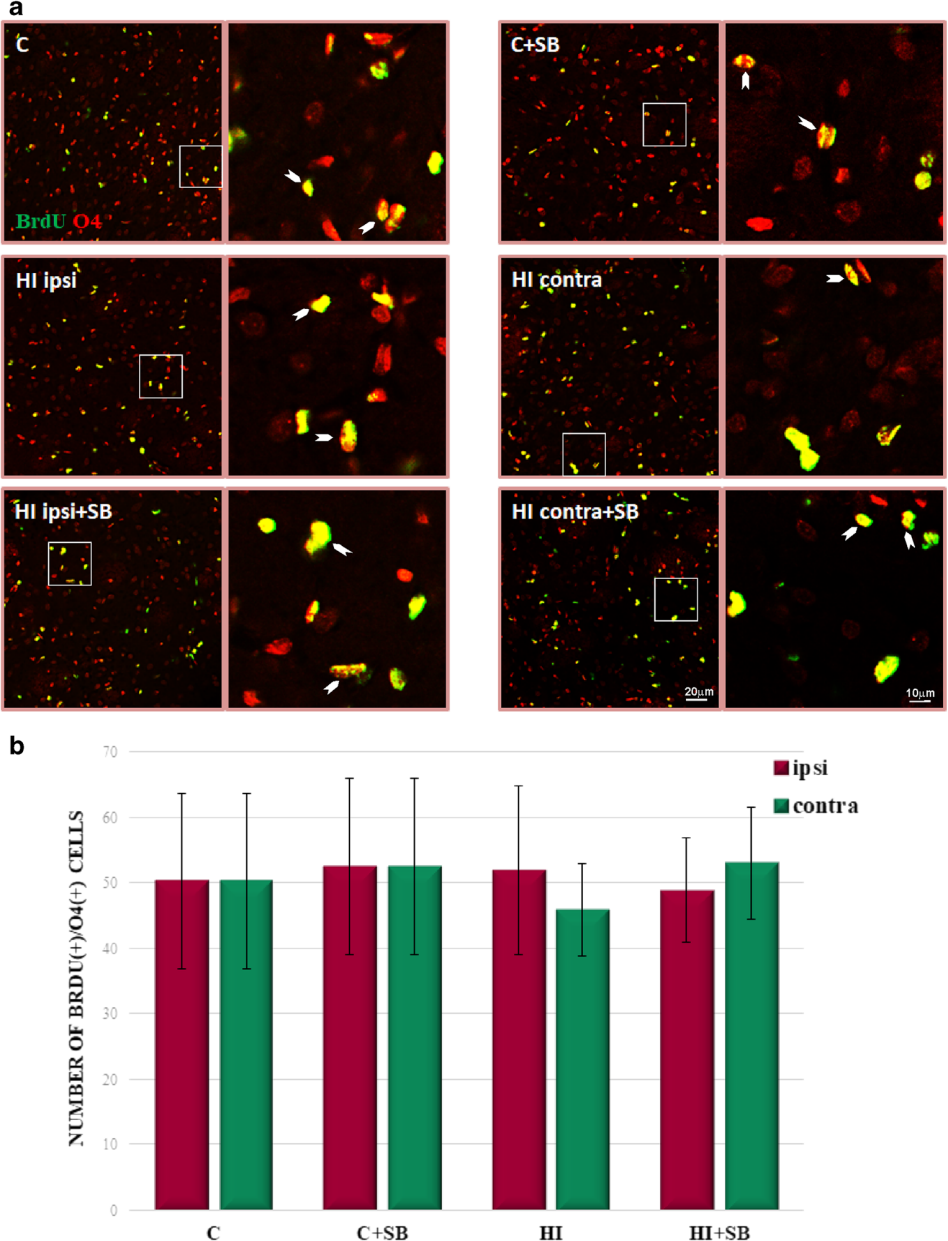
Sodium butyrate does not influence the number of nonmyelinating oligodendrocytes expressing O4 in the striatum after HI. Neonatal HI was induced at postnatal day 7. SB was administered directly after the onset of HI and for five consecutive days. BrdU was administered daily for three consecutive days starting 4 days after the onset of HI. Brain sections from control animals and from animals 28 days after HI were double-labeled with anti-BrdU antibody (green) and the nonmyelinating oligodendrocyte marker—O4 (*red*). **a** Confocal photomicrographs show double-labeled cells in the striatum (*arrowheads*). Enlargements present areas marked in rectangles. **b** Number of BrdU/O4-positive cells quantified in the striatum area (0.36 mm^2^). The values are mean ± SD from five animals per group. One-way ANOVA and Bonferroni test did not indicate significant differences between experimental groups. Abbreviations: *C*—control, *ipsi*—ipsilateral, *contra*—contralateral

In order to evaluate the differentiation of newly produced cells to oligodendroglial phenotype, we carried out double fluorescent studies for BrdU and specific markers. To address the question whether SB stimulates oligodendrogenesis within the SVZ area, we analyzed the number of proliferating oligodendrocyte precursor cells (OPCs) as well as more mature oligodendrocytes in the sham and HI animals at 2 and 4 weeks of recovery (P21 and P35, respectively). In control animals, we identified only a few BrdU-labeled cells co-stained with anti-NG2 within the SVZ (Fig. 8). The number of oligodendrocyte progenitor population between the investigated groups occurred to be not significantly different; however, a clear decreasing tendency, particularly in the ipsilateral side, was observed. Furthermore, neither HI alone nor HI together with SB treatment altered the number of nonmyelinating oligodendrocytes (BrdU/O4-positive) determined 28 days after the injury (P35) (Fig. 9). It should be added that numerous populations of BrdU/NG2 (Fig. 10) and BrdU/O4 (Fig. 11) cells were also identified within the striatum, but their number in each experimental group was very close to control.

### Microglia/Macrophage Response to Neonatal Hypoxia–Ischemia

**Fig. 12:**
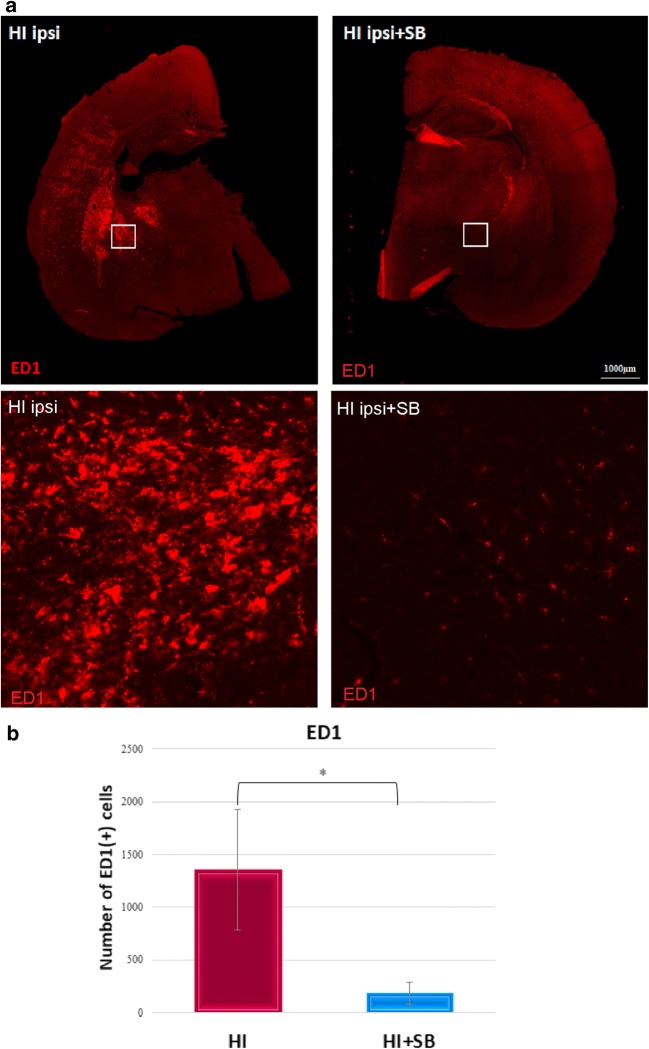
Sodium butyrate reduces microglial/macrophage cell number in the rat ipsilateral hemisphere after HI. Neonatal HI was induced at postnatal day 7. SB was administered directly after the onset of HI and for five consecutive days. Sections from SB-treated as well as untreated ipsilateral rat hemispheres were labeled with ED1 (marker for microglia and macrophages) antibody 14 days after HI. **a** Confocal photomicrographs of ipsilateral hemispheres show a large number of ED1-stained cells after HI and marked reduction after SB treatment. Enlargements present areas marked in rectangles. **b** Number of ED1-positive cells quantified in the whole ipsilateral hemisphere. The values are means ± SD from five animals per group. One-way ANOVA and Bonferroni test indicate significant differences between investigated groups, *p < 0.05. Abbreviation: *ipsi*—ipsilateral

**Fig. 13:**
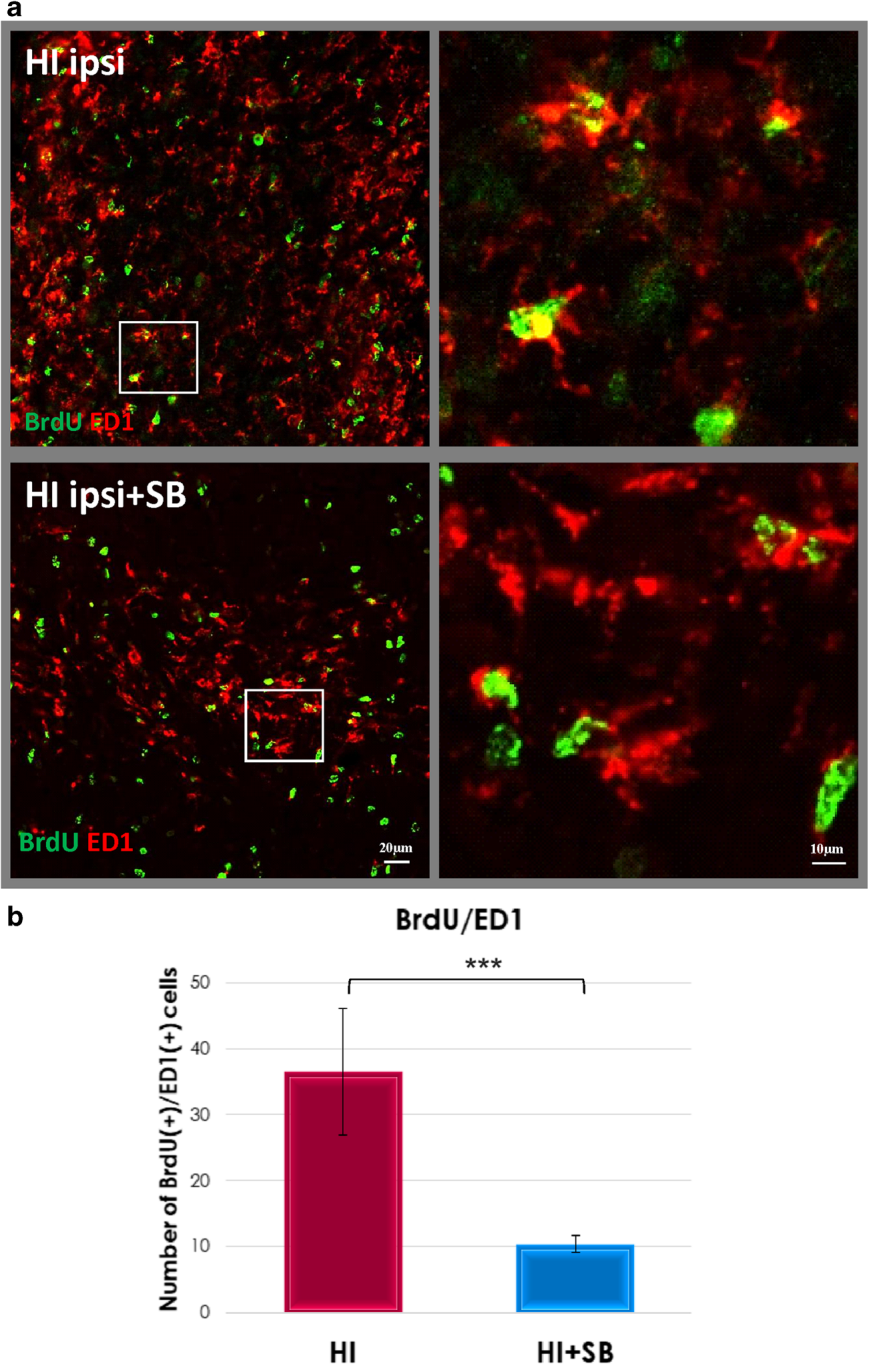
Sodium butyrate diminishes the generation of microglial/macrophage cells in the ipsilateral hemisphere after HI. Neonatal HI was induced at postnatal day 7. SB was administered directly after the onset of HI and for five consecutive days. BrdU was administered daily for three consecutive days starting 4 days after the onset of HI. Sections from SB-treated as well as untreated ipsilateral rat hemispheres were double-labeled with anti-BrdU antibody (*green*) and ED1 antibody (marker for microglia and macrophages) (*red*) 14 days after HI. **a** Confocal photomicrographs of brain sections show double-labeled cells (*yellow*). Enlargements present areas marked in rectangles. **b** Number of BrdU/ED1-positive cells quantified in the ipsilateral hemisphere area (0.36 mm^2^). The values are mean ± SD from five animals per group. One-way ANOVA and Bonferroni test indicate significant differences between investigated groups, ***p < 0.001 Abbreviation: *ipsi*—ipsilateral

Based on several studies suggesting a strong association of HI with inflammation, we examined the density of activated microglial cells labeled for ED1 staining in both brain hemispheres. As shown in Fig. 12, the ipsilateral side exhibited a large number of microglia/macrophage cells assessed by ED1 antibody at 14 days after HI. Most cells were round shaped with thick processes. They were scattered throughout the entire cortex and striatum. Contrary, these cells were not seen either in the contralateral side or in age-matched sham-operated animals. Sodium butyrate treatment significantly suppressed the number of ED1 expressing cells to 15% of vehicle-treated animals in the ligated side (*p* < 0.05) (Fig. 12). The decrease in the number of ED1-positive cells after SB administration to HI-injured animals correlated with a reduction (by about 70%) in newly generated microglia/macrophages, estimated by quantification of labeled BrdU/ED1 cells (*p* < 0.001) (Fig. 13).

**Fig. 14:**
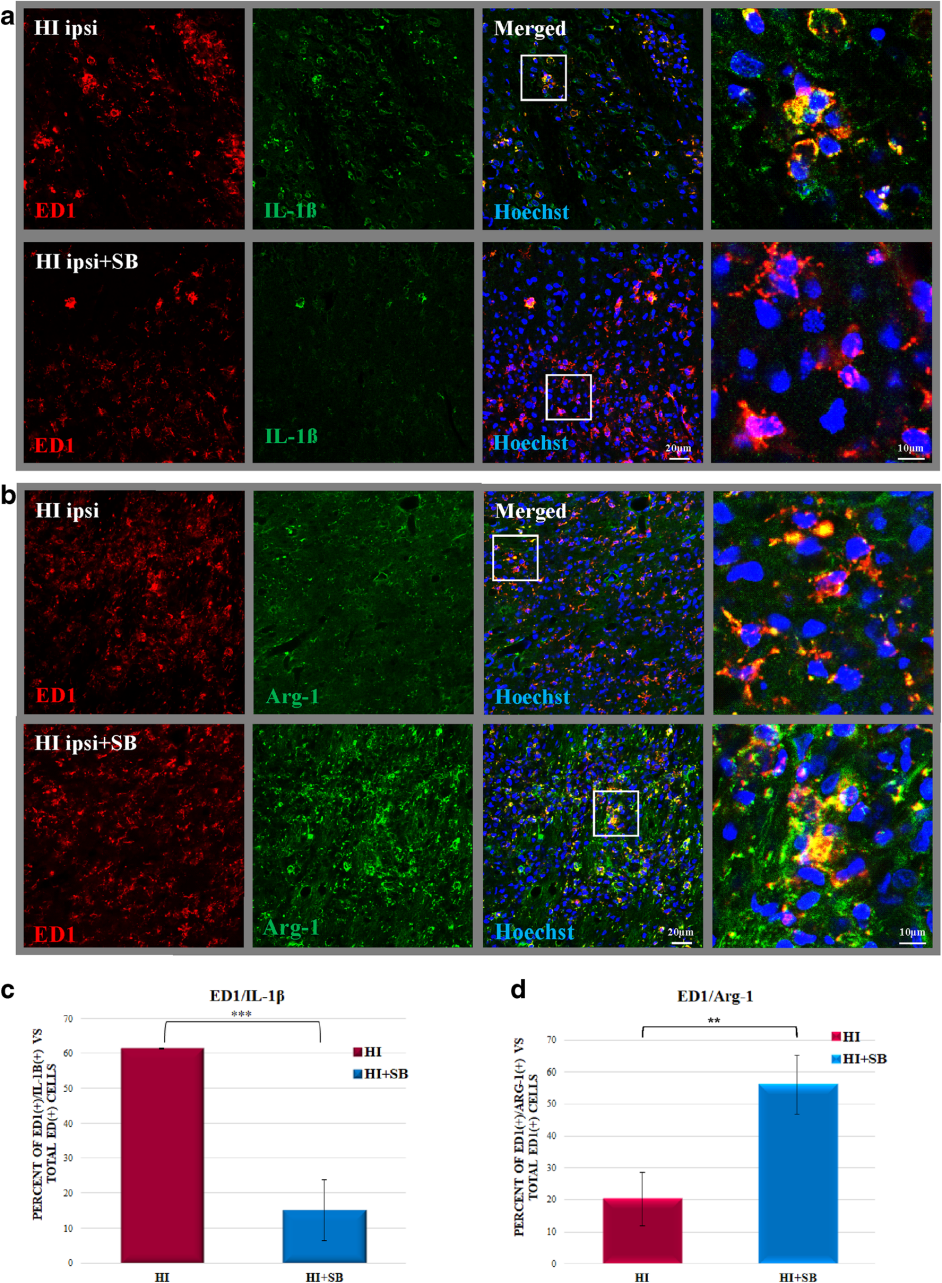
Sodium butyrate promotes the polarization of microglia from M1 to M2-like phenotype after HI. Neonatal HI was induced at postnatal day 7. SB was administered directly after the onset of HI and for five consecutive days. Sections from ipsilateral hemispheres 14 days after HI were stained for anti-ED1 antibody (*red*); and for anti-IL-1β, marker for M1 phenotype (*green*) (**a**); and for arginase-1 (Arg-1), marker specific for M2 phenotype (*green*) (**b**). Nuclei were labeled with the Hoechst dye (*blue*). Enlargement of areas marked in rectangles shows respective double-labeled cells (*yellow*). Number of IL-1β/ED1-positive cells (**c**) and Arg-1/ED1-positive cells (**d**) quantified in the ipsilateral hemisphere area (0.36 mm^2^). The values are means ± SD from five animals per group. One-way ANOVA and Bonferroni test indicate significant differences between investigated groups, **p < 0.01 and ***p < 0.001. Abbreviation: *ipsi*—ipsilateral

In the next step, we examined the effect of SB administration on the polarization of microglia from M1 to M2-like phenotype 14 days after HI. To address this, we carried out double staining with IL-1β antibody coupled with ED1 for the identification of activated proinflammatory M1 phenotype and ED1/arginase-1 for anti-inflammatory M2-like phenotype (Fig. 14). Two weeks after HI, the majority of ED1-positive cells expressed IL-1β in the cortical and striatal region of the ipsilateral hemisphere, with only a small part of cells stained positively with ED1/Arg-1. The administration of SB after HI led to a marked (about 4-fold) decrease in the number of cells presenting M1 phenotype of microglia (*p* < 0.001; ipsilateral vs ipsilateral with SB) with concomitant enhancement (above 2.5-fold) of cells stained with ED1/Arg-1 specific for M2 type (*p* < 0.01 ipsilateral vs ipsilateral with SB).

### The Effect of SB on the Acetylation of Alpha-Tubulin

**Fig. 15:**
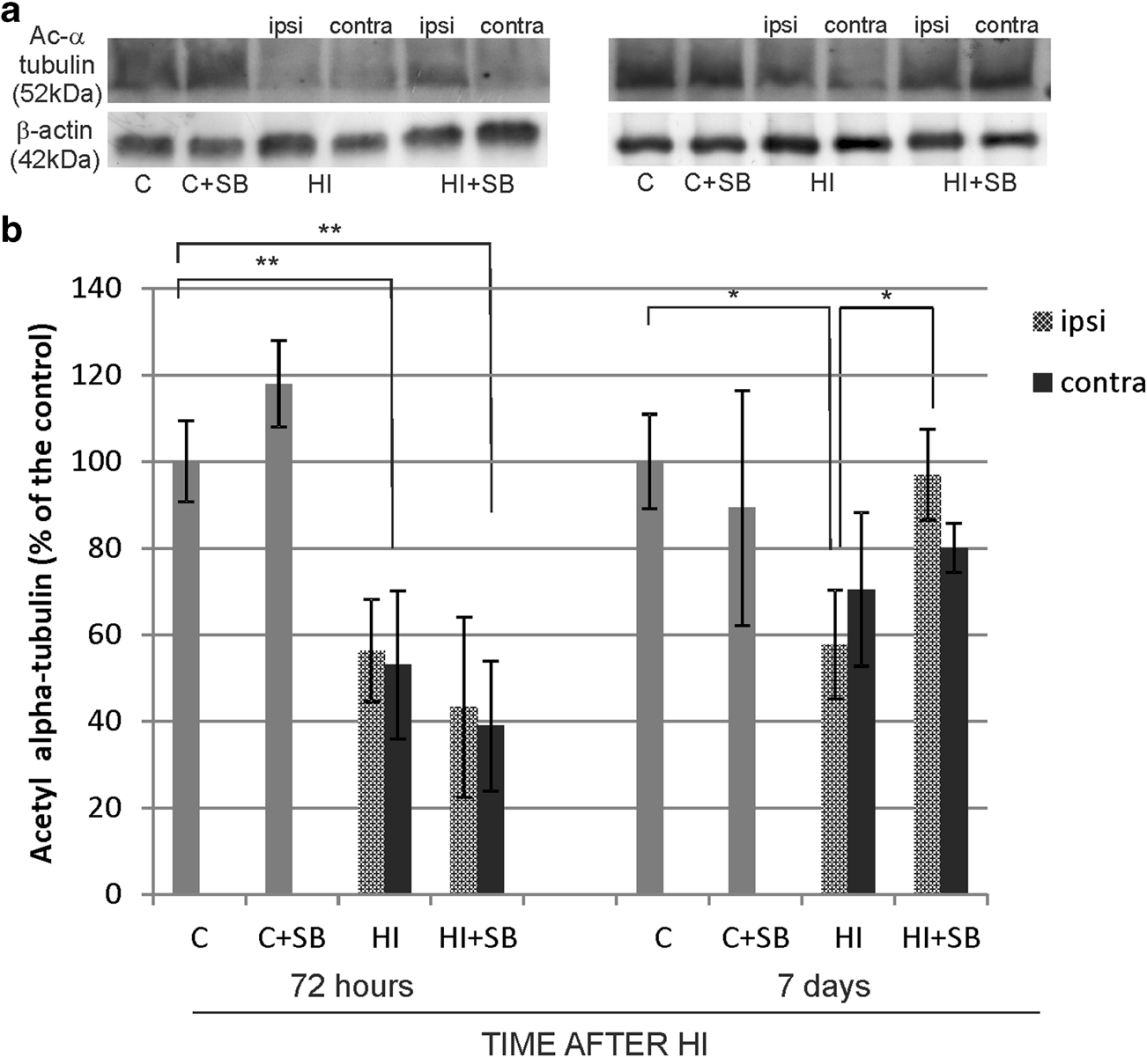
Sodium butyrate affects alpha-tubulin acetylation after neonatal hypoxia–ischemia. **a** Representative immunoblot of acetylated alpha-tubulin 72 h and 7 days after HI. The intensity of each band was quantified and normalized in relation to beta-actin. **b** Bar graph represents statistical analysis of densitometric data presented as percent of control value from indicated experimental groups. The values are mean ± SD from five animals per group and time point that were assessed in three independent experiments. One-way ANOVA and Bonferroni test indicate significant differences, *p < 0.05; **p < 0.01; Abbreviations: *C*—control, *ipsi*—ipsilateral, *contra*—contralateral

As our previous report showed that SB administration leads to increased acetylation of the histone H3 only in control animals, in the current study, we focused on alpha-tubulin, another HDAC substrate. Figure 15 shows the respective representative immunoblots probed with a specific antibody together with densitometric analysis. At 72 h after HI, the immunoreactivity of acetylated alpha-tubulin decreased in both brain hemispheres, compared to control (*p* < 0.01) independent of SB administration. The prolongation of postischemic time to 7 days resulted in a noticeable decrease in alpha-tubulin acetylation only in the ipsilateral hemisphere, by about 36% compared to the control (*p* < 0.05). SB treatment led to restoration of acetylated alpha-tubulin in the damaged side to the control value (*p* < 0.05 ipsi vs ipsi + SB). At the same time, the immunoreactivity of alpha-tubulin in the contralateral hemisphere did not show significant changes.

### Contribution of Neurotrophins to Sodium Butyrate-Induced Neurogenesis

According to the data indicating involvement of neurotrophins in promoting neurogenesis during brain development, we sought to investigate whether the effect of SB on the generation and differentiation of new cells found in the current study might be associated with changes of selected neurotrophins. As our previous results showed the effect of SB on mature BDNF protein 7 days after HI, in the present work, we aimed to assess the expression of pro-BDNF—precursor of mature brain-derived neurotrophic factor, which can stimulate distinct cell signaling pathways. Moreover, we also analyzed the changes in the level of another neurotrophin—NGF, and activation of ligand-receptor signaling. For this purpose, brain hemispheres were probed for total and phosphorylated TrkB receptor as well as receptor p75^NTR^.

#### Effect of Sodium Butyrate on Pro-BDNF

**Fig. 16:**
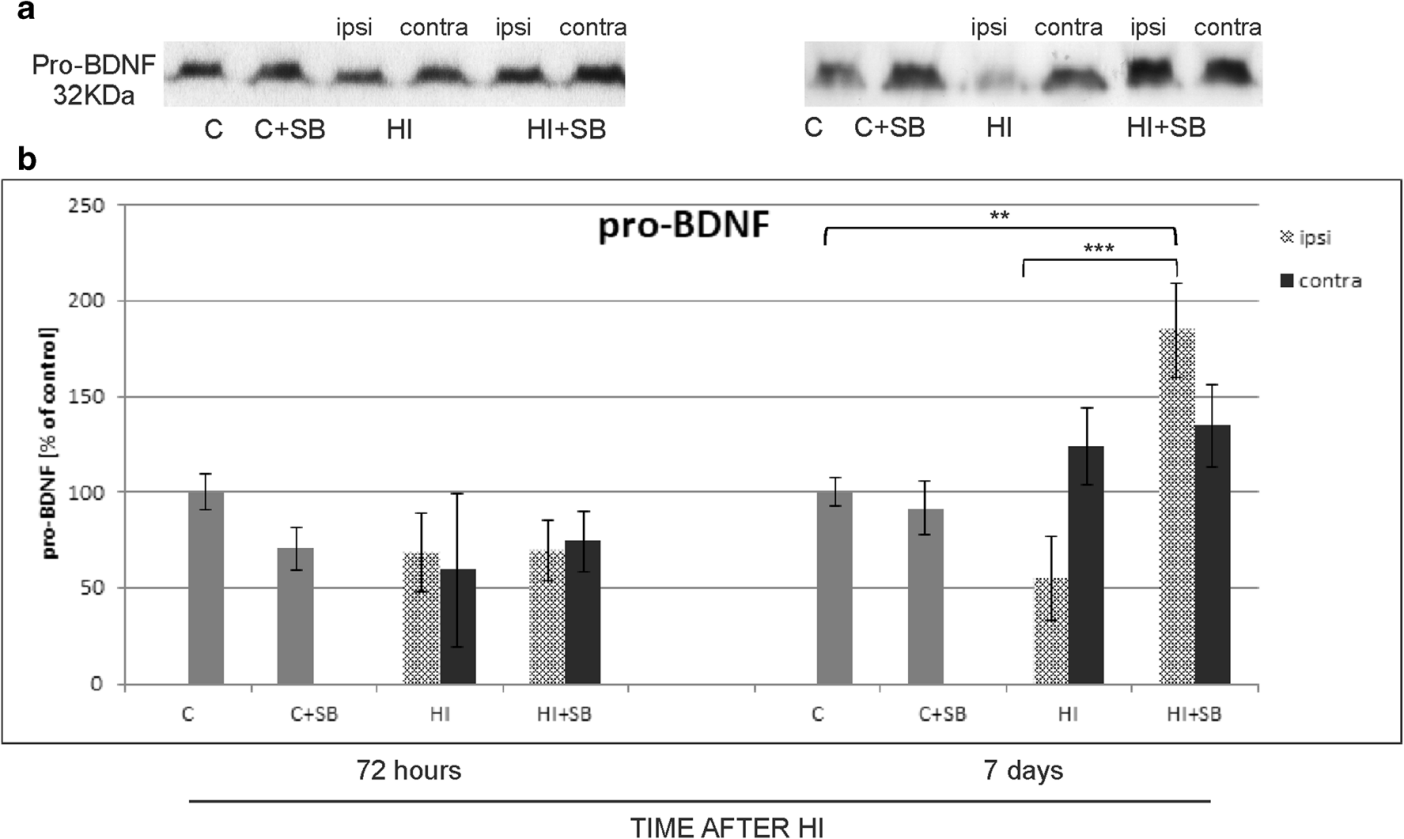
Effect of sodium butyrate on the expression of pro-BDNF after HI. **a** Representative immunoblots show the expression of pro-BDNF protein 72 h and 7 days after HI. The intensity of each band obtained by respective Western blotting was quantified and normalized in relation to β-actin. **b** Bar graph represents the statistical analysis of densitometric data presented as percent of control value from indicated experimental groups. The values are means ± SD from five animals per group and time point assessed in three independent experiments. One-way ANOVA and Bonferroni test indicate significant differences between investigated groups, **p < 0.01 and ***p < 0.001. Abbreviations: *C*—control, *ipsi*—ipsilateral, *contra*—contralateral

The results of immunoblot densitometry revealed increased level of pro-BDNF in the ipsilateral (hypoxic–ischemic) hemisphere after administration of SB. The significant elevation of pro-neurotrophin was observed 7 days after the insult (*p* < 0.001), compared to the untreated injured side (Fig. 16). Unexpectedly, 72 h postinjury, the expression of pro-BDNF presented almost the same value in all investigated groups, likewise as already mentioned in BDNF.

#### Effect of Sodium Butyrate on Nerve Growth Factor

**Fig. 17:**
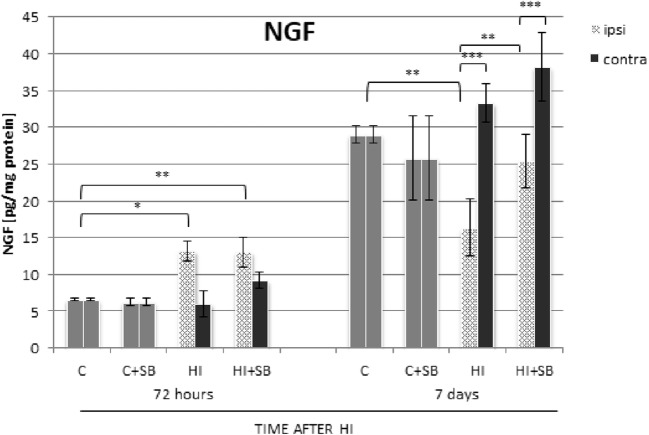
Effect of sodium butyrate on the expression of NGF after HI. Bar graph shows statistical analysis of NGF protein level assayed by ELISA. The values are means ± SD from five animals per group and time point assessed in triplicates. One-way ANOVA and Bonferroni test indicate significant differences between investigated groups, *p < 0.05, **p < 0.01, and ***p < 0.001. Abbreviations: *C*—control, *ipsi*—ipsilateral, *contra*—contralateral

In response to HI, NGF presents a different pattern of changes, compared to pro-BDNF (Fig. 17). The increase in NGF expression was noticed at 72 h of recovery in the ipsilateral side and was not dependent on the presence of SB. A marked reduction of NGF protein immunoreactivity was observed in the HI side at 7 days after the insult (*p* < 0.01 ipsilateral vs contralateral); however, it returned to control value after exposure to SB. In the contralateral hemisphere, the NGF level remained close to the value presented by the sham control throughout the entire experiment.

### Effect of Sodium Butyrate on Neurotrophin Receptors

In the next stage, the expression of specific BDNF receptor—TrkB and its phosphorylated form, as well as low affinity receptor p75, was evaluated at 72 h and 7 days after HI. To address this issue, we investigated whether SB treatment triggers any preferential activation of the receptor.

#### Effect of Sodium Butyrate on TrkB Receptor

**Fig. 18:**
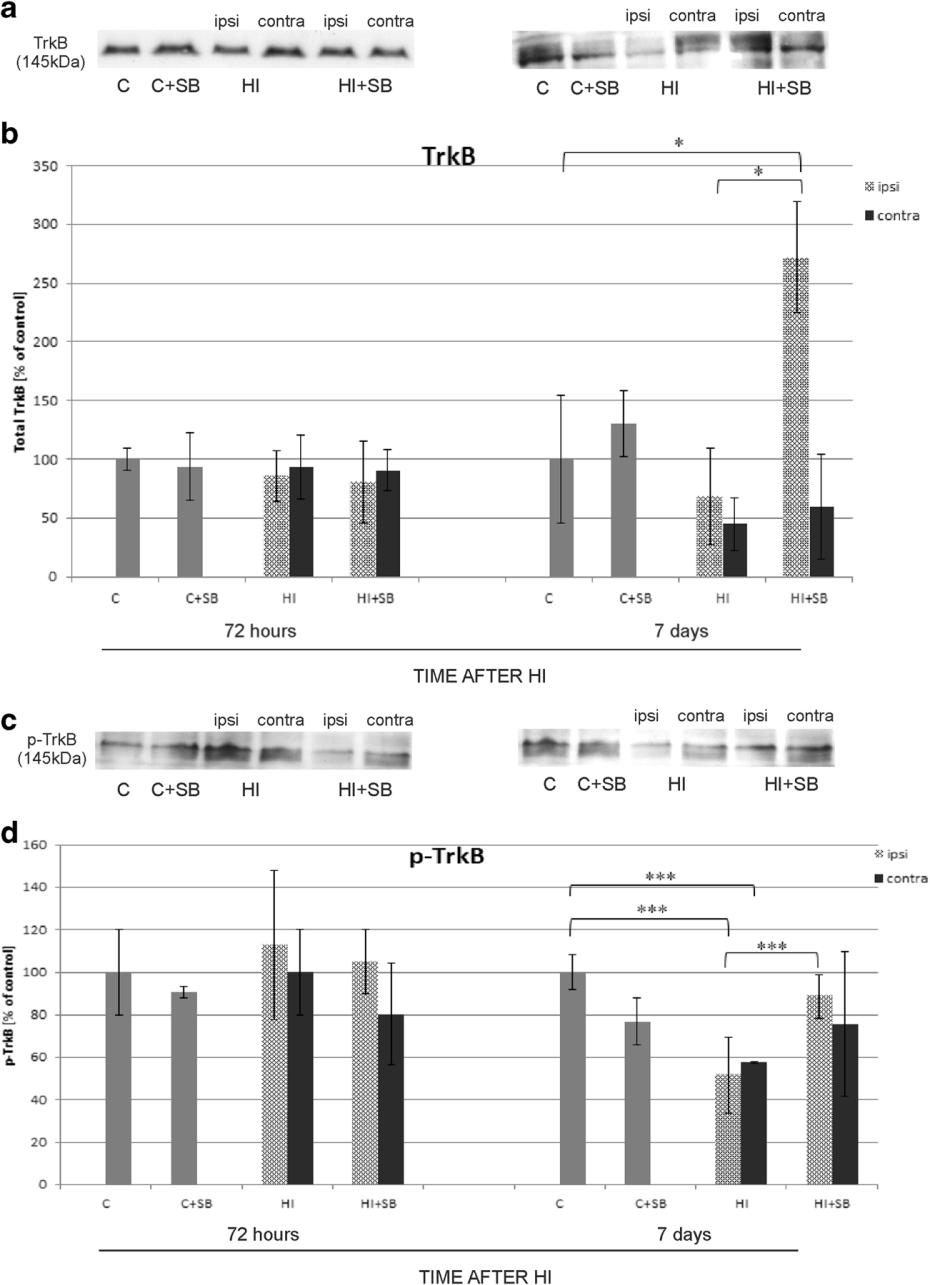
Sodium butyrate increases the level of TrkB and phospho-TrkB in the ipsilateral hemisphere 7 days after HI. Representative immunoblots of total TrkB (**a**) and phospho-TrkB (**c**) protein. The intensity of each band obtained by respective Western blotting was quantified and normalized in relation to β-actin. Bar graphs (**b** and **d**) represent statistical analysis of densitometric data presented as percent of control value from indicated experimental groups. The values are means ± SD from five animals per group and time point assessed in three independent experiments. One-way ANOVA and Bonferroni test indicate significant differences between investigated groups, *p < 0.05, ***p < 0.001. Abbreviations: *C*—control, *ipsi*—ipsilateral, contra—contralateral

To examine whether the SB-induced changes in pro- and mature BDNF are accompanied by changes in receptor expression, the total TrkB protein and its phosphorylated form (phospho-TrkB) were assessed after HI and drug treatment. The levels of protein were detected by estimation of immunoreactive band densities at 145 kDa. All data was compared to the respective control. We did not detect difference from the control level expression of TrkB at 72 h of recovery. A statistically significant effect of SB was noticed only in the ipsilateral hemisphere at 7 days after HI, when the immunoreactivity of the total receptor protein reached a value about 2-folds higher than the control value (*p* < 0.05) (Fig. 18a, b).

The analysis of the immunoreactivity level expressed by the phosphorylated/active form of the receptor revealed no changes in experimental groups at 72 h. Significant alterations in the level of phospho-TrkB were observed after 7 days. The expression of phospho-TrkB in both hemispheres of HI animals decreased to 50% of the respective control (*p* < 0.001). After SB treatment, the level of the phosphorylated receptor returned to control value in the ipsilateral side (*p* < 0.001) (Fig. 18c, d). It is worth pointing out that at this time of recovery, the reduction of phospho-TrkB after HI coincides with the level of total TrkB protein. Similarly, a correlation can be observed between total TrkB and phosphorylated TrkB in the ipsilateral hemisphere after SB treatment, when an increase and return to control value is seen.

#### Effect of Sodium Butyrate on Receptor p75^NTR^

**Fig. 19:**
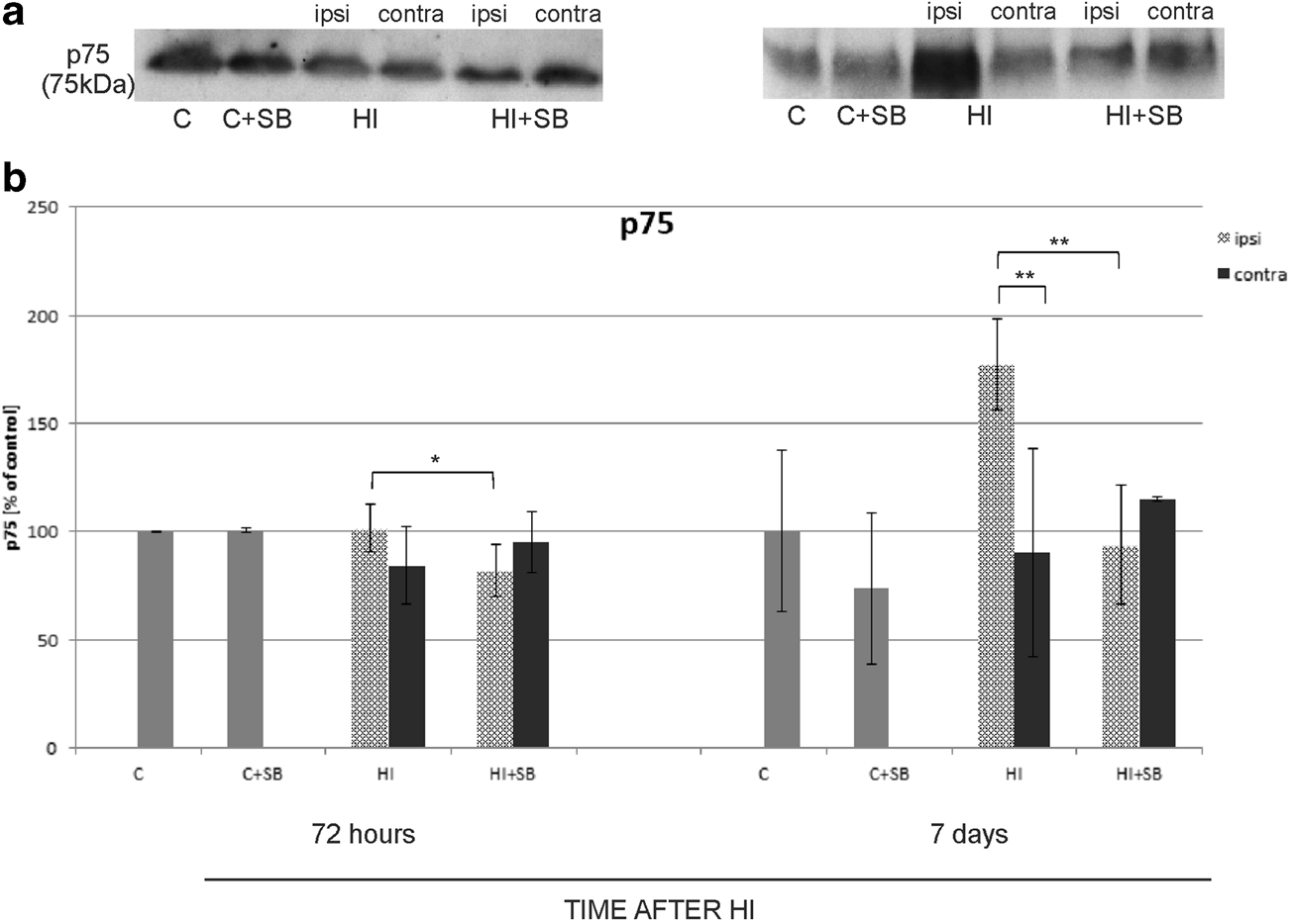
Sodium butyrate decreases the expression of p75^NTR^ in the ipsilateral hemisphere after HI. **a** Representative immunoblots of p75 protein. The intensity of each band obtained by respective Western blotting was quantified and normalized in relation to β-actin. Bar graph (**b**) represents statistical analysis of densitometric data presented as percent of control value from indicated experimental groups. The values are means ± SD from five animals per group and time point assessed in three independent experiments. One-way ANOVA and Bonferroni test indicate significant differences between investigated groups, *p < 0.05 and **p < 0.01. Abbreviations: *C*—control, *ipsi*—ipsilateral, *contra*—contralateral

Finally, in the same set of samples, we determined the influence of SB on the expression of p75 receptor. Brain tissue samples were blotted with corresponding antibody and the estimated band immunoreactivities are presented as percentage of the respective control. The effect of the insult alone (without SB treatment) was seen after 7 days and was exhibited by an increase in p75 immunoreactivity, almost 2-folds when compared to the control value. After SB treatment, all values returned to sham level (*p* < 0.01 ipsilateral vs ipsilateral with SB). It is worth noting that the effect of SB was also seen at an earlier time of recovery with a significant decrease (by about 20%) in p75 protein expression in the ipsilateral hemisphere 72 h after injury (*p* < 0.05 ipsilateral vs ipsilateral with SB) (Fig. 19).

### The Effect of SB on the Expression of Protein Kinases—ERK and Akt

Binding of neurotrophins to their receptors initiates a signaling cascade that promotes cell survival through activation of Ras–MAP and PI3K/Akt pathways. Therefore, to evaluate whether the changes in neurotrophins are accompanied by alteration in their downstream effectors, we analyzed the expression of ERK and Akt kinases, as well as their phosphorylated forms.

#### Protein Kinase ERK

**Fig. 20:**
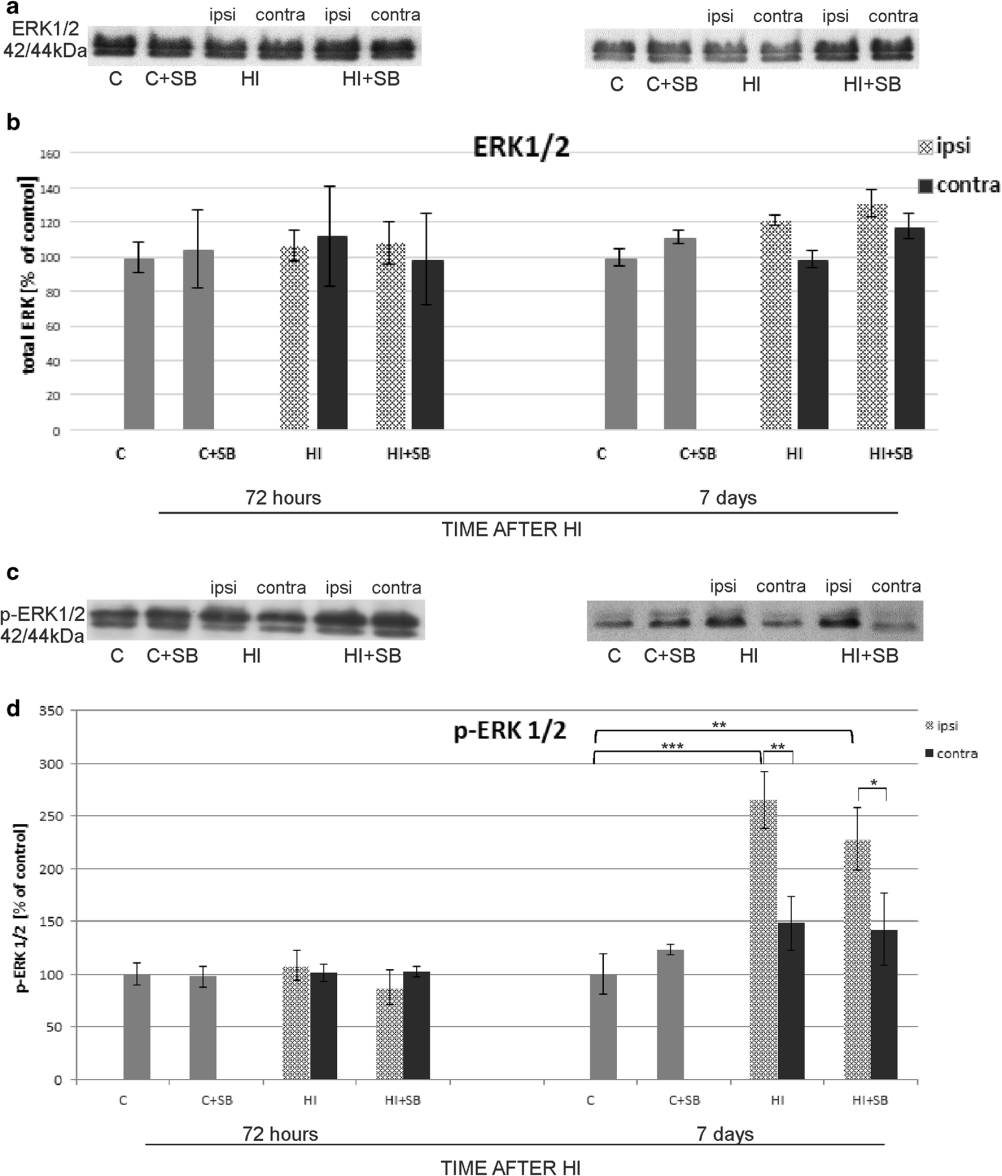
Effect of sodium butyrate on the expression of ERK1/2 and phospho-ERK1/2 in the brain after HI. Representative immunoblots of total ERK1/2 (**a**) and phospho-ERK1/2 (**c**) protein. The intensity of each band obtained by respective Western blotting was quantified and normalized in relation to β-actin. Bar graphs (**b** and **d**) represent statistical analysis of densitometric data presented as percent of control value from indicated experimental groups. The values are means ± SD from five animals per group and time point assessed in three independent experiments. One-way ANOVA and Bonferroni test indicate significant differences between investigated groups, *p < 0.05, **p < 0.01, and ***p < 0.001. Abbreviations: *C*—control, *ipsi*—ipsilateral, *contra*—contralateral

The ERK and phospho-ERK levels were determined by evaluating the band densities at 42/44 kDa. Figure 20 shows representative immunoblots with antibodies specific to total ERK1/2 protein, as well as to phospho-ERK1/2, together with densitometric analysis. Due to insufficient separation of samples, the data from two bands presenting total ERK, as well as phospho-ERK (kDa42/44), were summarized and are presented as a percentage of the respective control. As indicated in Fig. 20a, b, the total ERK expression in the analyzed experimental groups demonstrated close to control values during the entire course of this study. However, detailed analysis allows us to state that 1 week postinsult, there is, although very subtle, a tendency toward elevation of ERK expression in the damaged hemisphere after HI independent of SB treatment.

Furthermore, the only significant change in phospho-ERK activity was noticed 7 days after the insult in the ipsilateral hemisphere (Fig. 20c, d). An increase after HI injury (*p* < 0.001 ipsilateral vs control), as well as, however less pronounced, in the HI group subjected to SB treatment was observed (*p* < 0.01 ipsilateral + SB vs control).

#### Protein Kinase Akt

**Fig. 21:**
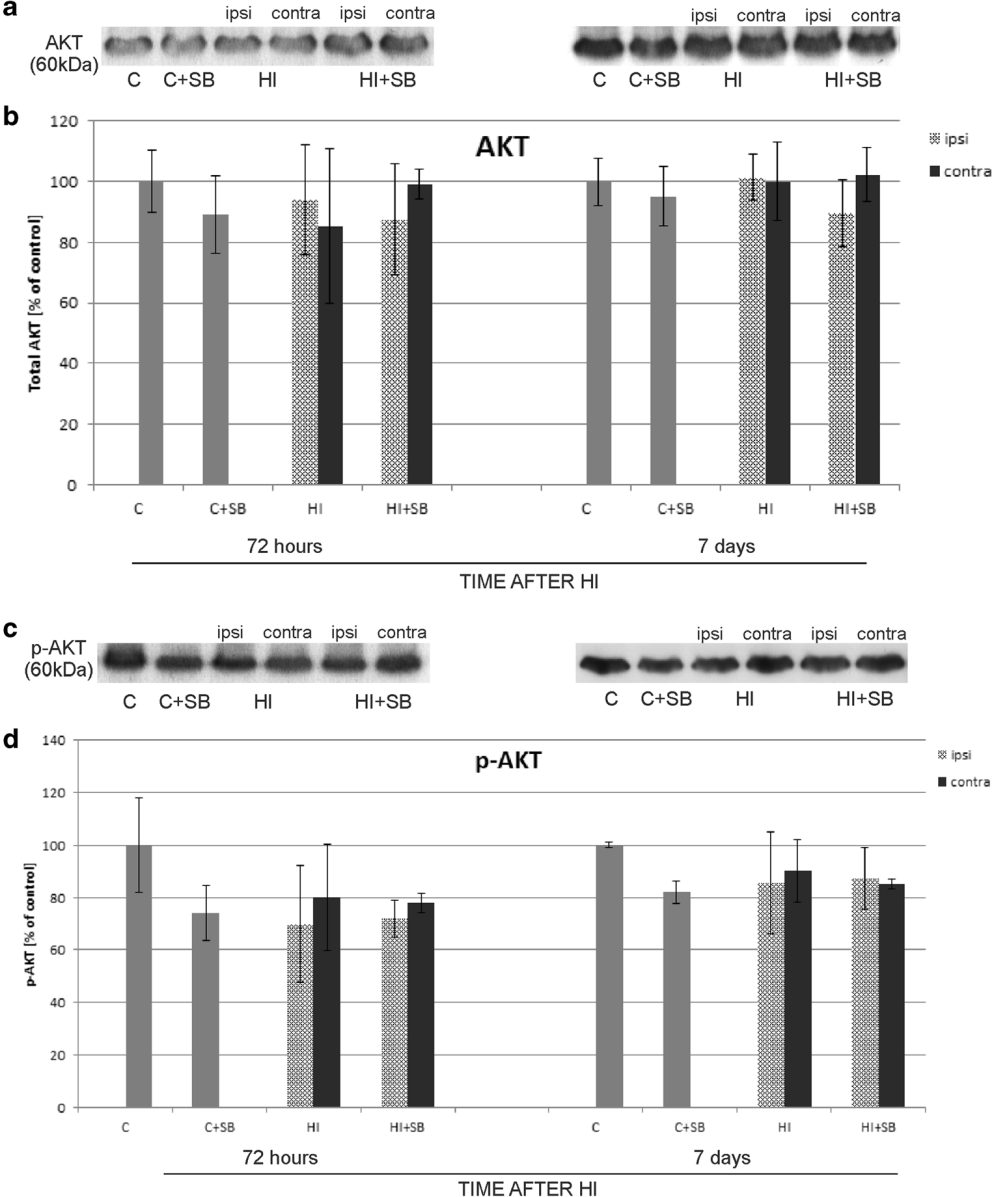
Sodium butyrate does not influence the expression of Akt and phospho-Akt in the brain after HI. Representative immunoblots of total Akt (**a**) and phospho-Akt (**c**) protein. The intensity of each band obtained by respective Western blotting was quantified and normalized in relation to β-actin. Bar graphs (**b** and **d**) represent statistical analysis of densitometric data presented as percent of control value from indicated experimental groups. The values are means ± SD from five animals per group and time point assessed in three independent experiments. One-way ANOVA and Bonferroni test did not indicate significant differences between investigated groups. Abbreviations: *C*—control, *ipsi*—ipsilateral, *contra*—contralateral

During the entire course of the experiment, the amount of total Akt protein did not show significant changes, as well as the phosphorylated form of this kinase remained on the level characteristic for the control group (Fig. 21).

### The Effect of SB on Transcription Factor CREB

**Fig. 22:**
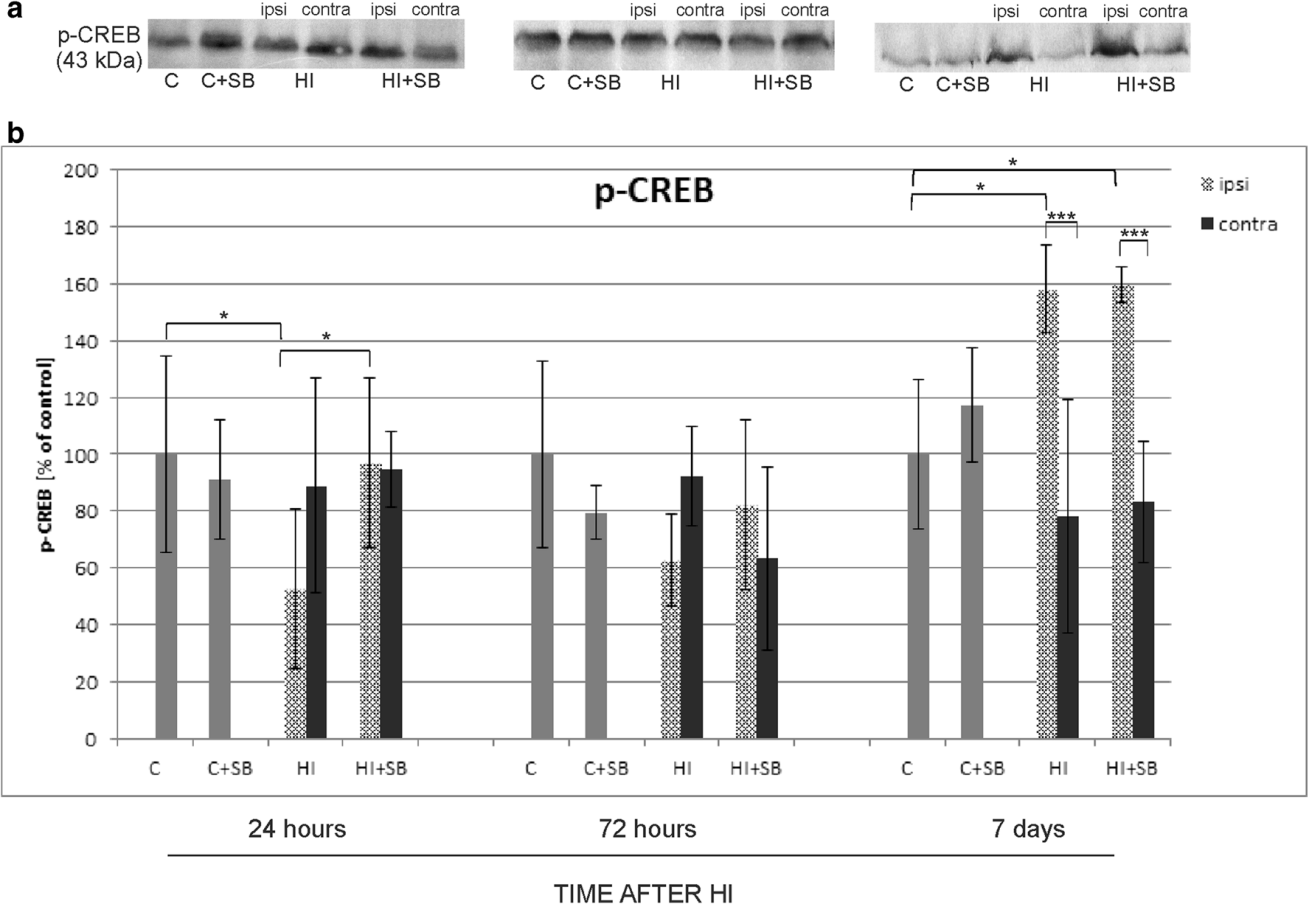
Effect of sodium butyrate on the expression of phospho-CREB in the brain after HI. **a** Representative immunoblots of phospho-CREB protein. The intensity of each band obtained by respective Western blotting was quantified and normalized in relation to β-actin. Bar graph (**b**) represents statistical analysis of densitometric data presented as percent of control value from indicated experimental groups. The values are means ± SD from five animals per group and time point assessed in three independent experiments. One-way ANOVA and Bonferroni test indicate significant differences between investigated groups, *p < 0.05 and ***p < 0.001. Abbreviations: *C*—control, *ipsi*—ipsilateral, *contra*—contralateral

In the following step, we examined the effect of SB on the expression of phosphorylated CREB (phospho-CREB Ser 133). Phospho-CREB is an important transcription factor downstream of the BDNF–TrkB signaling pathway. The WB assay data, followed by densitometric analysis, showed that 1 day after HI the response of phosphorylated CREB to the insult was associated with a marked decrease of protein immunoreactivity in the ipsilateral hemisphere compared to the control (*p* < 0.05) (Fig. 22). Administration of SB restored the expression level to sham value. At 7 days after the injury, the expression of phospho-CREB in the HI hemisphere increased to about 160% of the respective control and was not further influenced by HDACi treatment. In the contralateral hemisphere, the immunoreactivity of this factor presented close to control values during the entire course of the experiment.

## Discussion

The present study shows that treatment with the histone deacetylase inhibitor—sodium butyrate—after neonatal HI stimulates neurogenesis in the ipsilateral subventricular zone. The effect of HDACi was associated with an expanded population of neuroblasts identified by double staining (BrdU/DCX) and restoration of loss from HI injury neuronal cells. In addition, the HDAC inhibitor also demonstrated anti-inflammatory effects manifested by the reduction of the proinflammatory microglia accumulation.

Neonatal hypoxia–ischemia sets up a cascade of pathophysiological processes that leads to the massive loss of neurons and severe neurological deficits, despite the stimulated neurogenic response subsequent to neonatal brain injury. A body of work has shown an increased number of proliferating neural progenitor cells residing in the ipsilateral SVZ in rodents (rats, mice) that give rise to neuroblasts that migrate into the ischemia-damaged structure [12, 13, 15]. However, in spite of these interesting findings, there remains much doubt regarding the repair capacity of injured brain because the expected long-lasting effect on neuron numbers is not observed [12, 13, 42]. However, in our study, quantitative analysis of BrdU labeling in tissue samples gained 2 weeks after the injury shows that the population of proliferating cells in the hypoxic–ischemic (ipsilateral) as well as in hypoxic only (contralateral) SVZ remains close to age-matched sham-operated animals. These results are in agreement with our previous findings related to the neurogenic DG subgranular zone [26] as well as with data published by Qiu et al. [43] and Scheepens et al. [44] in mice subjected to HI or asphyxia, respectively. The unchanged level of the population of dividing cells may be due to a maximal rate of cell proliferation in the chosen stage of brain development, and then, it cannot be upregulated any further neither by HI insult nor epigenetic manipulation. It may be also probable that the pool of progenitors residing in the SVZ might be preferentially protected against ischemic depletion. It is worth noting that the subventricular zone was spared histologically by HI insult, whereas some brain areas (cortex, hippocampus) in the same conditions showed severe tissue damage. Interestingly, similar observations were described by Hayashi et al. [12] and Scheepens et al. [44]. The above data is to some extent in contrast to the results of Ong et al. [13] showing the altered size and shape of the ipsilateral SVZ. The obvious disparity could be due to the differences in experimental conditions including the strains of rodents and the severity of the insult, the majority being related to hypoxia time, which may affect the extent of brain damage and thus undoubtedly contribute to the variability in the number of BrdU-positive cells among the analyzed animals.

The unchanged intensity of proliferation in the hypoxic–ischemic SVZ found in the present study corresponds with the number of new neuroblasts (BrdU/DCX-positive). Otherwise, exposure to SB for 5 days following the onset of the insult appeared to increase the neuroblast population in the ipsilateral SVZ 14 days after HI and, importantly, enhanced the number of neuronal cells (BrdU/NeuN-positive) during the following 2 weeks (28 days after the injury). Collectively, this finding increases the possibility that new cells may supplement the lost neurons in the damaged area and hence participate in brain recovery. This prediction is reinforced by the fact that SB treatment decreased the cerebral damage by preventing severe atrophy or brain asymmetry as we already reported [26]. However, the effect of SB on the new neuron generation after HI, although significant compared either to the control or to the ipsilateral SVZ, has not been as robust as the stimulation rate of double-labeled BrdU/DCX cells. Hence, a subset of DCX-positive cells after the transient phase of upregulation at 14 days post-HI does not survive the process of maturation. It is consistent with data suggesting that most newly generated neuronal precursors derived from SVZ neurogenesis die before becoming mature cells [8, 15, 45]. Nevertheless, there are some reports indicating that SB, as well as other HDAC inhibitor treatments, induces cell differentiation and may contribute to long-term beneficial effects in adult rodents [32, 46].

Although cell migration has not been studied in this work in detail, in contrast to others [13, 15, 42, 47], we did not notice a direct evidence for the streaming of new neuroblasts from the immature SVZ toward the postischemic striatum. However, the current results demonstrate a sustained proportion of DCX-positive cells within the striatum area with only single cells presenting co-staining with BrdU. It cannot be excluded that neuroblasts that incorporated BrdU could have divided for an enough amount of time to dilute their BrdU content below the threshold of immunocytochemical detection. The other possibility is that some newborn neuronal progeny become postmitotic before the mitotic label administration and hence could not be detected. The distribution of neuroblasts in the HI group resembled that of controls.

In contrast to the report by Ong et al. [13], we identified newly generated neuronal cells (BrdU/NeuN-positive) within the striatum at 28 days after HI (35 PND). The newborn neurons were mostly pronounced in a zone adjacent to the SVZ. It may be supposed that new striatal neurons originate from local neural progenitors, possibly detached from the SVZ during maturation. Unexpectedly, after HI insult, the number of these new neuronal cells was close to the age-matched control. This remains in agreement with the studies showing that a small population of striatal neuroblasts in the ischemic brain can survive and mature into neurons [8, 48]. Unexpectedly, there was no effect of SB treatment on the generation of new neuronal cells in the striatum.

It is worth pointing out that only a few papers have presented the neuroprotective action of HDACis in immature brain, and those addressing the impact of HDAC inhibitors upon postinjury neurogenesis remain particularly limited [23–25, 49]. Moreover, the aims of the abovementioned studies did not always include at the same time the evaluation of maturation of proliferating cells into neurons with other cell types.

During the course of our study, we examined the response of oligodendrocyte progenitor population to HDACi. Immunofluorescent microscopy detected a consistently low level of double-stained (NG2/BrdU) glial progenitor cells in the SVZ. Moreover, in contrast to reports showing particular vulnerability of OPCs to neonatal insult [12, 50, 51], in our experiments, their number, analyzed at 14 days of recovery, was not significantly different from the control. However, detailed analysis of confocal imaging indicates a slight tendency of it to decrease, more pronounced in the ipsilateral side. It also occurred that sodium butyrate did not affect their number.

Likewise, the markers attributed to more advanced stages of oligodendrocyte maturation (Brdu/O4-positive cells) seem to be similarly distributed in this neurogenic area in the control as well as in injured animals at 4 weeks after HI, treated or untreated with SB. It might be due to the ongoing active gliogenesis process which is known to proceed most intensely during both the perinatal period and the first postnatal weeks in rats even after HI conditions [52, 53]. It may be supposed that the existing pool of cells is sufficient for sustaining endogenous homeostasis.

SVZ-derived oligodendrogenesis originates from a small fraction of C cells generating OPCs which migrate radially out of the SVZ into the overlaying white matter and cortex and probably take part in brain repair where oligodendrocytes are destroyed (for review, see [54]). Importantly, our study showed a stable level of BrdU/NG2 as well as more mature BrdU/O4 cells in the striatum. Nevertheless, the origin of OPCs requires further investigation.

However, during the course of this study, we did not investigate the generation of terminally differentiated oligodendrocytes. As an undisturbed level of MBP and PLP proteins was observed after HI in our previous studies, it may suggest the efficiency of the ongoing myelination [55].

It is worth pointing out that there are some intriguing data indicating that administration of the HDACi—valproic acid—during the first 10 postnatal days impairs oligodendrocyte differentiation manifested by significant hypomyelination and delayed expression of late differentiation markers [56]. On the other hand, Fleiss et al. [23] using a model of lipopolysaccharide-sensitized hypoxia–ischemia found that TSA, the common inhibitor of HDAC, did not affect the insult-reduced oligodendrocyte number, that could be observed by Olig-2 staining. Altogether, the effect of HDACis on oligodendrogenesis in immature brains remains unclear.

One of the most important findings obtained in the current work is that that the neurogenic effect of SB seen in the ipsilateral hemisphere was associated with reduction of HI-induced neuroinflammation at 14 days of recovery. This finding remains generally in consistence with those reported previously that show deacetylase inhibitors (VPA, TSA, and SB) to be efficacious neuroprotectants in adult cerebral injury models of stroke, resulting in a marked reduction in microglia number, suppression of their activation, and inhibition of inflammatory markers, which in turn lead to improved neuropathological outcome [18, 32, 46]. As demonstrated in the current study, sodium butyrate robustly diminished the enhanced generation of microglial cells (BrdU/ED1-positive) in the ipsilateral hemisphere at 14 days after the insult. Furthermore, the majority of ED1+ cells present a positive reaction with an established marker of M2 microglia phenotype, arginase-1, mostly pronounced in the SB-treated rats. The neuroprotective effect of SB expressed by regulation of the microglial inflammatory response via downregulating the expression of proinflammatory mediators and upregulating anti-inflammatory factors was confirmed recently in an experimental model of MCAO [57]. The beneficial role of M2 microglia after brain damage may be also connected with BDNF secretion [58–61]. It was evidenced that administration of exogenous microglia increases the expression of this factor in the ischemic hippocampus, which might lead to a neurotrophin-dependent protective activity in damaged neurons [59].

Our previous results showed that SB treatment of rats after HI induced a significant increase in the number of activated ED1-positive cells in the damaged ipsilateral hemisphere 6 days after the insult [62]. However, the majority of ED1+ cells presented co-staining with arginase-1 in SB-treated rats. Probably, in an early time after hypoxia–ischemia, SB administration induces microglial activation and promotes polarization from M1 to M2 phenotype. The prolongation of postischemic time to 14 days resulted in the decrease of ED1+ cell number after SB treatment, which may be related to microglia deactivation. Altogether, in the light of our previous as well as current findings, it may be speculated that SB facilitates the conversion of M1 to M2 phenotype leading to anti-inflammatory signaling and, by this, keeps microglia from acquiring a proinflammatory phenotype and, in consequence, prevents tissue damage, such as that found in models of AD, MS, and neurodegeneration [63–65]. This prediction may be reinforced by the parallel decrease in the number of ED1/IL-1β-positive cells observed during the present study. Transition of microglia during recovery from the proinflammatory (M1) to immunomodulatory and neurotrophic response (M2) [66, 67] and then maintenance of endogenous neurogenesis may play a key role in attenuation of brain damage [68]. To confirm the role of microglial reaction to HI injury in the developing brain and, in particular, to define the time course of M1 to M2 polarization, further studies will be needed.

At present, it is rather impossible to precisely outline the molecular mechanisms directly linked to the observed beneficial effects of sodium butyrate after neonatal HI. HDACi treatment after ischemia induced in adult rodents leads to a marked upregulation of histone acetylation, a virtue of HDAC inhibitory activity [18, 46]. In line with this, Fleiss et al. [23] demonstrated an increased acetylation level of H3 and H4 after TSA treatment in LPS/HI female mice pups. In contrast, our previously reported results, obtained by Western blot assay, may suggest that the effect of SB administered after neonatal HI induced in 7-day-old rats is not achieved through increased acetylation of histone H3 [26]. Considering the fact that histone deacetylase isoforms have been shown to regulate acetylation of a plethora of nonhistone proteins, the possibility that the neuroprotective effect of HDAC inhibitors may influence a diverse array of targets cannot be ruled out [69, 70]. One of the supposed mechanisms of HDACi action may be facilitation of axonal transport by returning the reduced acetylation level of alpha-tubulin [38]. The significant decrease of acetylated tubulin after HI insult observed in the current study probably indicates axonal cytoskeletal breakdown and disruption of axonal transport, the well-recognized features of HI brain injury [71–73]. The results of our study show a protective effect of SB treatment on the alpha-tubulin acetylation level in the ipsilateral side, in agreement with already reported action of TSA on the rescue of axonal transport and locomotor behavior [74].

Importantly, anterograde transport of neurotrophins and then their terminal release may constitute a crucial means of neurotrophic support in which loss may account for some neurodevelopmental changes and neuronal death [75–77]. Therefore, it is clear that HDACi could act as a key regulator of trophic factor provision. Trophic factor secretion is postulated as the primary or supplementary mechanism engaged in neurogenesis in the SVZ after stroke and brain trauma [78–80]. Hence, this study presents a thorough investigation of SB influence on neurotrophins—pro-BDNF and NGF—as well as on signaling proteins in parallel with the highest proliferation rate. The previous study performed in our laboratory showed that SB administration stimulates the expression of BDNF in the ipsilateral hemisphere at 7 days of recovery after neonatal HI [26]. The resembling response of the total TrkB receptor as well as its phosphorylated form in the ipsilateral vs contralateral hemisphere observed in our work may imply a supporting role of BDNF–TrkB signaling in SB-induced protection and neurogenesis. The engagement of this pathway in mediating HDACi-induced proliferation and differentiation in the ischemic adult rodents has been previously confirmed [32, 33]. Thus, at least, this mechanism mediated by BDNF underpinning neuroprotection after SB treatment in neonates is supposed to be one of those commonly reported in adult cerebral studies.

In contrast to mature BDNF binding to the TrkB receptor, pro-BDNF activates selectively its high affinity receptor p75, leading mainly to neuronal damage in vitro and in vivo [81–85]. The elevated expression of p75 receptor at 7 days after HI injury returned to control level after HDACi application. This remains in line with a similar reaction of p75 receptor in the presence of HDACi in a model of TBI [86]. The above responses of p75 receptor to SB application may also indicate the beneficial action of SB.

Several published data point out to the controversial role of p75 receptor in regulating neuronal fate, specifically in potentiating the effects of Trk receptors aimed at neuronal survival. Furthermore, it was found that p75-deficient mice display reduction of adult neurogenesis [87]. On the other hand, numerous subsequent studies have highlighted the role of p75 overexpression in neuronal and glial cell death in adults after brain injury [84, 88]. However, the biological consequences of maintaining the p75 receptor on the control level in the HI hemisphere after SB treatment are not possible to define. Nevertheless, the increased expression of pro-BDNF found in our study and the concomitant decrease of p75 receptor protein may indicate that pro-BDNF rather serves as a precursor of BDNF than a biologically active protein preferably binding to the p75 receptor. Therefore, the elevated levels of this factor likely promote survival and neurogenesis.

In addition, SB application also prevented the HI-induced reduction of NGF protein in the ipsilateral HI side at 7 days of recovery. The beneficial action of NGF enhancement by pretreatment with SB was reported in other brain injuries [89–91]. It is suggested that the survival response of nerve growth factor is mediated by the TrkA receptor, whose activation may be associated with suppression of the JNK kinase activity, the mediator of hypoxia-induced neuronal cell death [92]. A probable criticism of the present study is that it was not possible to detect the level of TrkA. Nevertheless, maintaining the expression of NGF on the control level in the presence of HDACi could imply that this response participates in neuroprotection.

Altogether, the enhancement of pro-BDNF, BDNF, and NGF expression following SB treatment may confer neuroprotection/neurogenesis. This is supported by reports showing that ICV administration of NGF or BDNF increases the number of new neurons and neuronal precursors generated in neurogenic zones of adult brains and protects against neuronal death [93–95].

The consequences of neurotrophin receptor binding result in the subsequent activation of specific intracellular signaling pathways such as PI3K/Akt and ERK, which lead to phosphorylation of transcription factor cyclic AMP-responsive element binding protein (CREB) involved in inducing prosurvival gene expression. The elevation of both factors—phospho-ERK as well as phospho-CREB—in the ipsilateral hemisphere at 7 days after HI remains the same after SB treatment. These findings fit to the assumption that in damaged cells ERK1/2 activation may act as an important defensive and pro-neurogenic mechanism [34, 36, 96–99]. The action of pERK1/2 may be due to induced expression of prosurvival genes via the transcription factor CREB [100, 101]. Thus, it may be postulated that the activation of phospho-CREB in response to HI may represent a cellular form of protection. Such speculation could be supported by previous work showing stimulation of CREB phosphorylation in adult ischemic animals [102–104]. Considering the probable mechanism of ERK activation after HI, nontranscriptional response, linked among other pathways to stimulation of BDNF–TrkB, cannot be excluded.

However, in our work, the expression of pERK1/2 and pTrkB at 7 days of recovery shows a temporal relationship only in the presence of SB. Thus, it seems possible that the mode of specific signaling pathway activation depends on the regulatory mechanism stimulated under certain conditions. ERK1/2 kinase activity can be activated/phosphorylated also in response to several other factors released by brain injury, such as pro-chemokines, cytokines, and oxidative stress [105–107]. The determination of the way of ERK activation needs further investigation. In contrast to the response of ERK kinase activity, kinase Akt remained on the control level during the entire course of the current study. Therefore, unexpectedly, it does not seem to be involved in neurogenesis after neonatal HI insult.

## Conclusion

Our present results demonstrate that sodium butyrate, an inhibitor of histone deacetylases, administered after neonatal HI, exerts a neurogenic/neuroprotective effect in the ipsilateral (hypoxic–ischemic) SVZ. SB-induced neurogenesis is associated with a marked inhibition of HI-induced inflammation expressed by transition of microglia to anti-inflammatory phenotype (M2). Considering the fact that neonatal HI triggers simultaneous processes due to expression and a release of factors resulting in neurogenesis and most of these processes may regulate each other, it is not possible to determine the precise mechanism of SB action responsible for the generation of new neurons. However, it is tempting to speculate that BDNF–TrkB signaling may contribute to the effects of SB after neonatal hypoxic–ischemic injury. Continuing research along this line may provide a better understanding of responses to the inhibition of histone deacetylases in the neonatal hypoxic–ischemic brain.
